# Enhancing Hydroxyl Metabolite Analysis through In Situ Derivatization-Enabled Desorption Electrospray Ionization-Mass Spectrometry

**DOI:** 10.1021/acs.analchem.5c04858

**Published:** 2025-11-14

**Authors:** Yen-Chu Lin, Guan-Yuan Chen, Ya-Jin Jheng, Hsiao-Wei Liao

**Affiliations:** † Department of Pharmacy, College of Pharmaceutical Sciences, 34914 National Yang Ming Chiao Tung University, No.155, Sec.2, Linong Street, Taipei 112304, Taiwan; ‡ Forensic and Clinical Toxicology Center, 33561 National Taiwan University, No.7, Zhongshan S. Rd., Zhongzheng Dist., Taipei 100225, Taiwan; § Department and Graduate Institute of Forensic Medicine, College of Medicine, 33561 National Taiwan University, No.7, Zhongshan S. Rd., Zhongzheng Dist., Taipei 100225, Taiwan

## Abstract

Unlocking the full potential of metabolomics hinges on significantly improving the detection of metabolites, particularly those containing hydroxyl groups, which often remain challenging to ionize. Our previous work established 2-(4-Boronobenzyl) isoquinolin-2-ium bromide (BBII) as a highly effective derivatization reagent for enhancing hydroxyl metabolite sensitivity in liquid chromatography–mass spectrometry. Building upon this, we herein introduce a novel and robust extension: BBII derivatization integrated with desorption electrospray ionization (DESI) for *in situ* hydroxyl metabolite detection. This innovative approach involves incorporating BBII directly into the DESI spray solvent, leading to significant sensitivity enhancement through instantaneous derivatization reaction. We demonstrate substantial signal increases ranging from 1.8- to 17.2-fold for hydroxyl metabolite reference standards, making previously undetectable compounds such as glucose, hexadecanol, and estradiol readily observable. When applied to mouse brain tissue sections for mass spectrometry imaging (MSI), BBII-DESI successfully revealed the distinct spatial distributions of representative hydroxyl metabolites, including glucose and cholesterol that are typically invisible to conventional methods. A key advantage of this methodology is the characteristic boron isotopic pattern of BBII-derivatized features, facilitating rapid and precise screening and identification. This BBII-assisted DESI strategy effectively unveils the “dark metabolome” of hydroxyl compounds, providing access to previously inaccessible metabolic information. Our method broadens the utility of ambient ionization techniques by enabling direct analysis of biological samples with minimal preparation, which is crucial for high-throughput applications. This advance offers substantial potential for accelerating biomarker discovery and disease diagnostics through direct visualization of metabolic alterations within native tissue environments, thereby marking a significant leap forward in spatial metabolomics applications for health and disease research.

## Graphical abstract

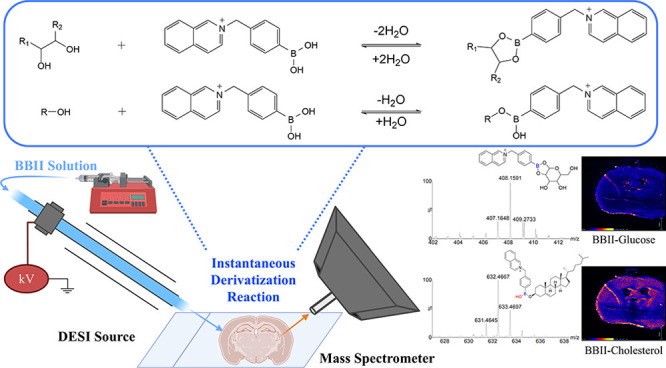

## Introduction

Unlocking the intricate world of small molecules, metabolomics stands as a pivotal field that aims to comprehensively profile metabolites (<1500 Da) within biological systems, providing profound insights into physiological states and disease mechanisms.^1–4^ Mass spectrometry (MS) is a cornerstone technology in this domain, providing the requisite high sensitivity and selectivity. This has established MS as an indispensable tool across diverse applications, including drug development, systems biology, biomarker discovery, and personalized medicine.^5–10^ In particular, untargeted metabolomics enables the unbiased analysis of complex metabolic networks, enabling the detection of unexpected or novel metabolite changes without prior knowledge of analyte identity.^11^

Mass spectrometry imaging (MSI) is a powerful technique that enables the visualization of various metabolites directly in tissue sections, preserving spatial context.^12^ Several ionization methods have been widely employed for MSI experiments, such as matrix-assisted laser desorption/ionization (MALDI), secondary ion mass spectrometry (SIMS), and desorption electrospray ionization (DESI).^13–15^ DESI, an ambient ionization technique, allows for the direct analysis of sample surfaces under atmospheric pressure, thereby eliminating the need for laborious extraction or preparation steps. This capability for real-time, spatially resolved chemical analysis positions DESI as a robust tool for diverse applications, including tissue imaging, drug localization studies, and on-site diagnostics.^16–18^ Despite the advanced capabilities of modern MS techniques in metabolomic analysis, the effective detection of certain metabolite classes, particularly polar and neutral hydroxyl-containing compounds such as sugars, polyols, phenols, and alcohols, remains a significant hurdle. To overcome these limitations, chemical derivatization is employed to introduce ionizable or permanently charged groups, which enhances MS signal intensity, analyte stability, and fragmentation efficiency.^19^ Boronic acid–based reagents have proven effective for selectively targeting cis-diol groups through reversible boronate ester formation, offering a valuable strategy for focusing on specific subsets of hydroxyl metabolites.^20–23^ Furthermore, on-tissue derivatization facilitates increased selectivity or sensitivity for poorly ionized metabolites while preserving their native spatial characteristics in biological samples.^24–26^ In reactive DESI, the spray solvent is modified to contain a derivatization reagent, enabling *in situ* derivatization to enhance specific analytes within the DESI process.^27^ Reactive DESI approaches targeting hydroxyl metabolites using boronic acid–based derivatization reagents have been previously explored.^28,29^ However, the comprehensive application of reactive DESI-MSI for hydroxyl metabolites remains insufficiently established. Specifically, to our knowledge, no reports exist on the analysis of diverse classes of hydroxyl metabolites using reactive DESI-MSI.

2-(4-Boronobenzyl) isoquinolin-2-ium bromide (BBII), a chemical labeling reagent originally developed for serum glucose detection, has recently been adapted for MALDI time-of-flight mass spectrometry (MALDI-TOF-MS) analysis.^30^ BBII’s key feature is a permanently charged quaternary ammonium group, which markedly enhances ionization efficiency, particularly in positive-mode MS. It also offers the advantages of rapid derivatization kinetics, avoiding time-consuming pretreatment, and exhibiting a distinct boron isotope pattern useful for the confident identification of labeled compounds. Our earlier work demonstrated BBII’s efficacy in derivatizing a diverse array of hydroxyl metabolites, including not only conventional diol groups but also alcoholic and phenolic hydroxyl groups, which substantially improved their MS detectability.^31^

In this study, we present BBII-assisted reactive DESI (BBII-DESI) as a novel strategy coupling real-time derivatization with ambient ionization. This approach enables the enhanced detection and imaging of hydroxyl metabolites directly from sample surfaces. We investigated two implementation strategies: (1) prespraying the BBII reagent onto the sample surface prior to DESI ionization and (2) incorporating BBII directly into the spray solvent. Key experimental parameters, including heated transfer line temperature, BBII concentration, spray incident angle, and capillary voltage, were systematically optimized to maximize derivatization efficiency and signal intensity. The enhanced analytical performance was validated using hydroxyl metabolite standards and subsequently applied to mouse brain tissue sections. The results demonstrate the utility of BBII-DESI in high-sensitivity, spatially resolved molecular imaging *in situ*, highlighting its potential for applications in biomarker discovery and disease diagnostics.

## Experimental Section

### Chemicals and Reagents

Acetonitrile (ACN, HPLC gradient grade) was purchased from Duksan (Ansan-si, South Korea). Diethyl ether (ether, ≥ 99.8%) was obtained from Honeywell (Charlotte, NC, USA). Deionized water (DIW) was prepared using the PURELAB Ultra system (ELGA LabWater, High Wycombe, UK). Unless otherwise specified, all other chemicals were purchased from MilliporeSigma (St. Louis, MO, USA).

The standard compounds selected for this study represented a range of biologically relevant hydroxyl compounds, including carbohydrates (glucose), long-chain alcohols (hexadecanol), steroids (cholesterol and estradiol), catecholamines (dopamine), nucleosides (adenosine), and pyridoxine. Stock solutions of each standard compound were prepared in 50% DMSO/ACN (v/v) at a concentration of 1 mg/mL and stored at 4 °C for later use. For the analysis, individual standard solutions were combined in a single reaction vial and diluted with ACN to obtain a final sample solution containing each standard compound at a concentration of 10 μg/mL.

The derivatizing reagent BBII (C_16_H_15_BNO_2_Br) was synthesized as previously described and stored as a dried product at 4 °C for later use.^30^ The synthesis was validated through mass spectrometry, confirming the molecular ion peak at *m*/*z* 264.1190.

### Instrumentation

BBII-DESI experiments and DESI imaging were performed in positive ion mode using a Synapt XS HDMS Q-TOF mass spectrometer equipped with a DESI ion source (Waters Corporation). The DESI source was operated in “sensitivity mode” for data acquisition. Prior to each experiment, the mass spectrometer was calibrated using leucine enkephalin with an ESI source. The optimized instrumental parameters comprised a sprayer-to-surface height of 0.5 mm, sprayer-to-MS inlet distance of 1 mm, spray incident angle of 72°, and heated transfer line temperature of 400 °C. The capillary voltage was maintained at 1.4 kV, while the sampling cone voltage was set to 40 V. Nebulizing gas (N_2_) pressure was regulated at 0.06 MPa.

For system optimization, the BBII solution (5 μg/mL, prepared in 75% ACN/water (v/v)) was delivered using a Pump 11 Elite Programmable Single Syringe Pump (Harvard Apparatus) at a constant flow rate of 3 μL/min. To optimize BBII-DESI conditions, a mixture of standard metabolites was deposited onto porous PTFE Printed Slides (Electron Microscopy Sciences). Aliquots (5 μL) of the standard mixture were manually spotted in a linear array on the PTFE-coated glass slide with interspot spacing of approximately 6 mm. All samples were allowed to air-dry at ambient temperature prior to analysis. In optimization experiments, the DESI spray was manually controlled to scan across four standard mixture spots at a translation speed of 100 μm/s. Each experimental variable was evaluated in triplicate to generate 4 × 3 data sets for statistical analysis.

Before each tissue analysis, we performed a two-step procedure to ensure system stability. First, we tuned the DESI source using a rhodamine 6G marker (*m*/*z* 443.2329, positive ion mode), which included optimizing the capillary voltage and nebulizing gas pressure to establish a stable spray and signal. Second, we verified the BBII solution’s reactivity by spraying it onto standard compounds to confirm adequate derivative generation and signal intensity. An Acquity H Class SM-FTN UPLC Sample Manager (Waters Corporation) was employed to deliver the BBII solution at a flow rate of 4 μL/min. The lateral spatial resolution for imaging was set to 25 μm with a stage translation speed of 50 μm/s. Mass spectral data for optimization experiments were acquired over a mass range of *m*/*z* 50–1000, while tissue imaging employed a range of *m*/*z* 100–1200.

### Tissue Sample Preparation

Brains from blank C57BL/6 mice were harvested following sacrifice and rinsed twice with phosphate-buffered saline (PBS) to remove residual blood. Excess moisture was carefully blotted, and the tissues were immediately snap-frozen in liquid nitrogen before being transferred to a – 80 °C freezer for storage. Coronal brain sections were prepared at – 18 °C using a CM3050 cryostat (Leica Microsystems, Wetzlar, Germany) at a thickness of 15 μm, without the use of optimal cutting temperature (OCT) compound. Sections were mounted directly onto glass microscope slides and stored at – 80 °C prior to DESI analysis.

### Data analysis

For optimization experiments, average spectra from each spot were obtained using MassLynx version 4.2 (Waters Corporation) and processed for statistical analysis. High-definition imaging (HDI) version 1.7 (Waters Corporation) was employed for imaging data acquisition, raw data processing, and image visualization. During DESI experiments, the *m*/*z* 264.1190 ion of BBII was used as a lock mass, and postacquisition correction was applied via the HDI software. Mass spectral data filtering for BBII-derivatized hydroxyl metabolites and compounds of interest was performed using a mass window of *m*/*z* 100–1000, with the number of most intense peaks set to 1000 and a mass tolerance of ± 0.02 Da. The identification of BBII-derivatized hydroxyl metabolites in mouse brain sections was accomplished through systematic screening for the characteristic boronic isotope pattern from total ion chromatogram and mass spectrometry images, specifically monitoring for the presence of an M-1 signal with approximately one-quarter the intensity of the base peak. The precursor ion *m*/*z* was extrapolated by subtracting BBII’s mass and adding back one or two molecules of H_2_O. Therefore, both monohydroxyl compound and diol compound possibilities were evaluated, resulting in two possible masses for the suspected features. Subsequently, both possible monohydroxyl and diol masses were cross-referenced with databases and literature to confirm metabolite identity. For annotation of unidentified features of interest, comprehensive metabolomic databases were utilized, including the Human Metabolome Database (HMDB), Metabolomics Workbench, LIPID MAPS Structure Database, and MaizeGDB.

## Results and Discussion

### Real-Time Derivatization of Hydroxyl Metabolites by BBII-DESI

Based on our previous work, BBII instantaneously reacts with hydroxyl-containing compounds, forming boronate esters. This derivatization is characterized by the elimination of one or two water molecules, yielding characteristic product ions corresponding to [M+BBII-H_2_O]^+^ and [M+BBII-2H_2_O]^+^, respectively (Figure 1A).^31^ Consequently, two implementation strategies were evaluated to explore the optimal conditions for BBII-DESI. In Method 1, the BBII reagent was presprayed onto a glass slide spotted with a standard mixture prior to the DESI analysis. Method 2 incorporated BBII directly into the spray solvent to facilitate an *in situ*, real-time derivatization reaction on the sample spots. As illustrated in Figure S1, both strategies successfully yielded the anticipated derivatized ions. However, Method 1 demonstrated inferior reproducibility and signal intensity compared to Method 2; notably, key analytes such as cholesterol and estradiol were poorly detected by Method 1. We hypothesize that the predeposition of BBII onto the sample surface may lead to an uneven distribution of analytes during the solvent drying process. Furthermore, implementing Method 1 in MSI workflows presents significant challenges in accurately controlling the amount of BBII solution applied, where excessive solution volume could potentially compromise the morphological integrity of tissue sections. In contrast, Method 2 facilitated uniform derivatization and bypassed the laborious pretreatment procedures associated with Method 1. Given its superior derivatization performance, greater operational simplicity, and seamless integration into existing MSI workflows, the strategy of incorporating BBII into the spray solvent (Method 2) was selected for all subsequent experiments (Figure 1B).

**Figure 1:**
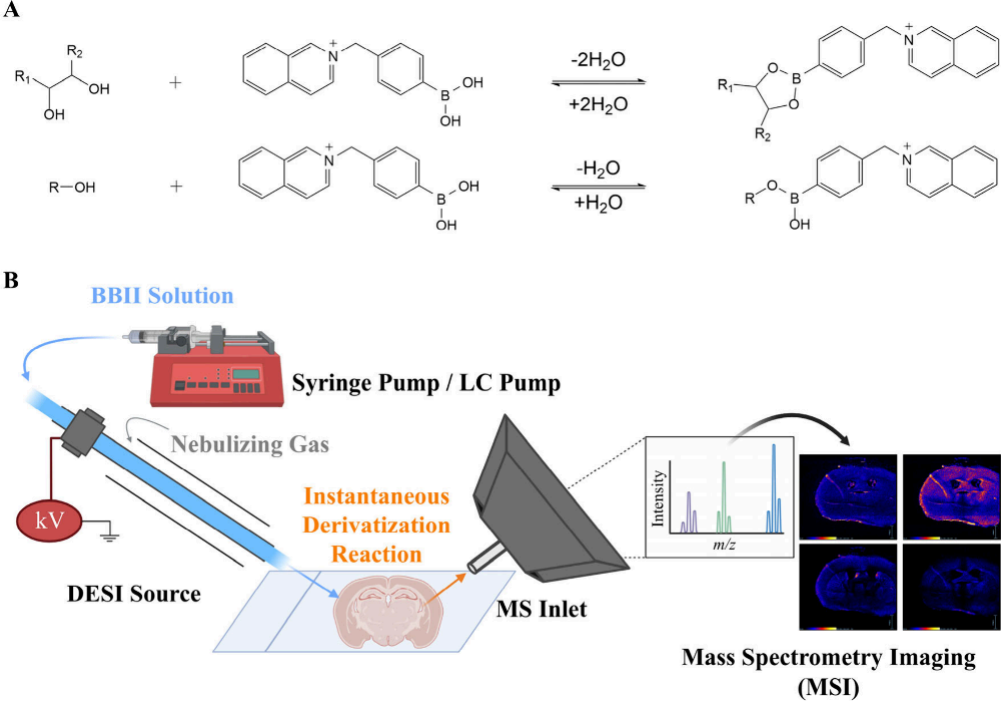
(A) Chemical derivatization reactions of BBII with diol and hydroxyl-containing compounds. (B) Schematic diagram of the BBII-DESI-MSI experimental setup.

The derivatization reaction mechanism was systematically evaluated using a representative panel of seven standard hydroxyl metabolites, encompassing diverse structural classes including catecholamines (dopamine), vitamins (pyridoxine), carbohydrates (glucose), fatty alcohols (hexadecanol), nucleosides (adenosine), and steroid hormones (estradiol, cholesterol). The analytical workflow involved depositing the compound mixture in a linear array, followed by DESI spray scanning across the dried analyte spots. Figure 2 provides comprehensive mass spectrometric evidence for successful derivatization, with each spectrum exhibiting the diagnostic boron isotope pattern. As demonstrated in Figure 2A, BBII displays characteristic peak pairs at *m*/*z* 264.1194 and 263.1228, corresponding to ^11^B­[BBII]^+^ and ^10^B­[BBII]^+^ isotopologues, respectively. These peaks exhibit the expected 1:4 intensity ratio with a mass difference of 0.9966 Da, consistent with the natural abundance of boron isotopes. Alongside these primary reagent ions, a minor signal at *m*/*z* 220.1126 was observed, corresponding to the loss of the boronic acid group. To confirm that this ion’s formation did not affect experimental reproducibility, we evaluated the reagent’s stability. First, data from a previously conducted 5-day stability test of the BBII solution (in 75% ACN/water) showed no accumulation of the *m*/*z* 220.1126 ion in the stored solution. This finding supports our hypothesis that *m*/*z* 220.1126 is a stable, in-source fragment generated during the DESI process, rather than a product of chemical degradation accumulating in the solvent over time. Second, to directly assess stability during prolonged MSI acquisitions, we investigated the signal behavior throughout an extended scan. We monitored the ratio of the *m*/*z* 220.1126 fragment to the primary BBII reagent signal, which remained constant and low (less than 10%) throughout the entire MSI process. This demonstrates that the relative levels of the active reagent and its in-source fragment are stable, ensuring reproducible derivatization conditions throughout the experiment. Figures 2B–H demonstrate that all derivatized hydroxyl metabolites retained this distinctive isotopic signature with comparable mass differences, confirming successful boronic ester formation across the entire metabolite panel.

**Figure 2:**
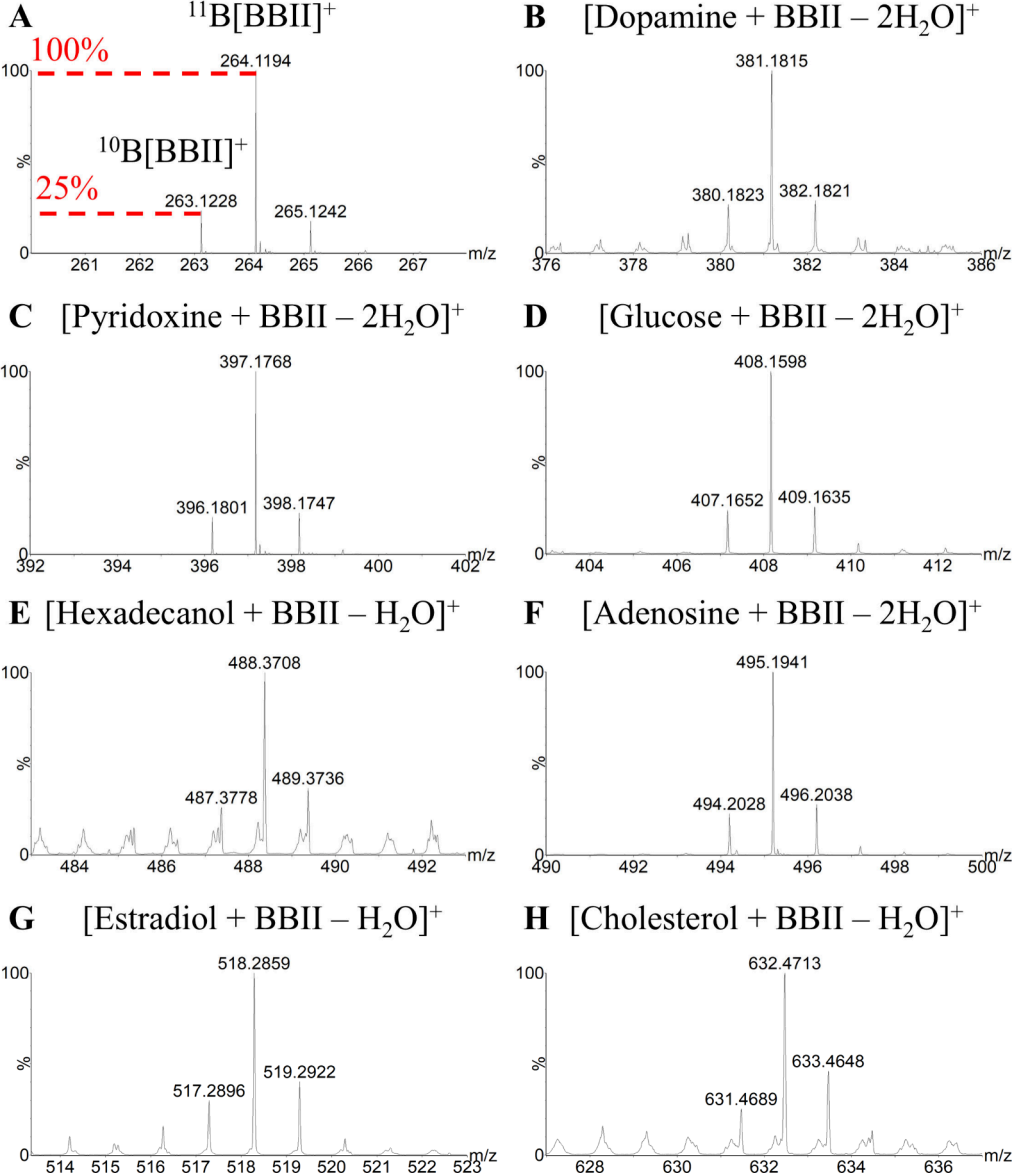
Mass spectrometric analysis of BBII reagent and its metabolite derivatives. (A) Isotopic pattern of BBII showing characteristic boron isotope distribution with ^11^B­[BBII]^+^ (*m*/*z* 264.1194) and ^10^B­[BBII]^+^ (*m*/*z* 263.1228). (B–H) Mass spectra of BBII-derivatized metabolites: (B) dopamine, (C) pyridoxine, (D) glucose, (E) hexadecanol, (F) adenosine, (G) estradiol, and (H) cholesterol derivatives, showing expected molecular ion peaks after derivatization reactions.

Metabolites containing vicinal hydroxyl groups, such as dopamine, pyridoxine, glucose, and adenosine, yielded derivatives corresponding to [M+BBII-2H_2_O]^+^. Conversely, hexadecanol, estradiol, and cholesterol, which lack vicinal hydroxyl groups, produced derivatives corresponding to [M+BBII-H_2_O]^+^ (Figure S2). In our previous study, glucose, pyridoxine, dopamine, and adenosine occasionally generated minor derivatives due to potential side reactions resulting from either boronic ester hydrolysis or incomplete derivatization processes where BBII interacted with a single hydroxyl group, manifesting as the [M+BBII-H_2_O]^+^ ion. However, under the BBII-DESI approach, only pyridoxine and adenosine exhibited a minor derivative at *m*/*z* 415.1879 and 513.2073 (Figure S3A, S3B), and the proportions of minor derivatives were 3.4% and 2.4%, respectively. Based on derivative signal intensities, pyridoxine demonstrated the highest response among all hydroxyl metabolites, approximately 10-fold greater than the others. This suggests that pyridoxine exhibits enhanced reactivity toward BBII derivatization, consequently increasing its propensity to generate minor derivatives. Notably, when the transfer line temperature was elevated, pyridoxine preferentially generated the derivative at *m*/*z* 397.1768 rather than *m*/*z* 415.1879, presumably because higher thermal conditions facilitate more efficient water evaporation and suppress the reverse reaction.

Notably, estradiol possesses two reactive hydroxyl groups: a phenolic group at C-3 and an alcoholic group at C-17. In our experiments, we exclusively observed a product corresponding to the mass of a single BBII-estradiol adduct, and no double-labeled derivatives were detected. While the specific reaction site was not definitively determined, we conducted experiments to confirm BBII’s reactivity toward both types of functional groups. Reactivity with alcoholic hydroxyls was previously demonstrated in our work with corticosterone, which only contains alcoholic hydroxyls on its steroid structure.^31^ To verify reactivity with phenolic groups, we tested BBII with hydroquinone, which contains only phenolic hydroxyls, and confirmed its successful derivatization (Figure S3C). These results demonstrate that BBII is capable of reacting with both phenolic and alcoholic sites, even though it forms only a single-site adduct with estradiol. Conclusive site localization would require further structural elucidation, such as tandem mass spectrometry to identify diagnostic fragments.

In summary, the BBII-DESI approach successfully enables real-time derivatization of diverse hydroxyl-containing metabolites under ambient conditions. Mass spectrometric analysis confirms high derivatization efficiency across structurally diverse compounds, with characteristic boron isotope patterns serving as diagnostic markers for successful boronic ester formation. This approach represents a significant advancement in ambient ionization techniques for hydroxyl metabolite analysis. By eliminating the requirement for additional sample pretreatment, BBII-DESI enables rapid, high-sensitivity detection directly from biological surfaces.

### Optimization BBII-DESI

To establish optimal conditions for BBII-DESI, critical experimental parameters including heated transfer line temperature, BBII concentration, spray incident angle, and capillary voltage were systematically optimized. The results, based on the average signal abundance from triplicate scanning of four standard mixture spots, provided insights into the efficacy of the optimized conditions. According to the BBII derivatization mechanism, boronic ester formation occurs concomitantly with dehydration. To enhance reaction efficiency, we anticipated improved performance at elevated temperatures, as higher thermal conditions would facilitate more efficient water evaporation and suppress the reverse reaction according to Le Chatelier’s principle. We systematically evaluated different heated transfer line temperature conditions to optimize the derivatization reaction. Figure 3A demonstrates the effect of temperature elevation on BBII-DESI efficiency. As temperature increases, hydrophobic metabolites, including hexadecanol, estradiol, and cholesterol, showed minimal impact on their signal abundance. However, hydrophilic metabolites, such as glucose, pyridoxine, and adenosine, exhibited enhanced derivatization efficiency at higher temperatures. This observation suggests that these hydrophilic metabolites, which form [M+BBII-2H_2_O]^+^ derivatives, are more significantly influenced by dehydration conditions, resulting in increased signal abundance as temperature is elevated. It is noteworthy that dopamine, despite being a hydrophilic metabolite, shows minimal temperature dependence, which may be attributed to its relatively lower derivatization efficiency compared to other metabolites. Compared to the 400 °C condition, the 450 °C condition showed marginal enhancement in signal abundance while exhibiting greater variability. Accordingly, a transfer line temperature of 400 °C was selected as the optimal derivatization condition.

**Figure 3:**
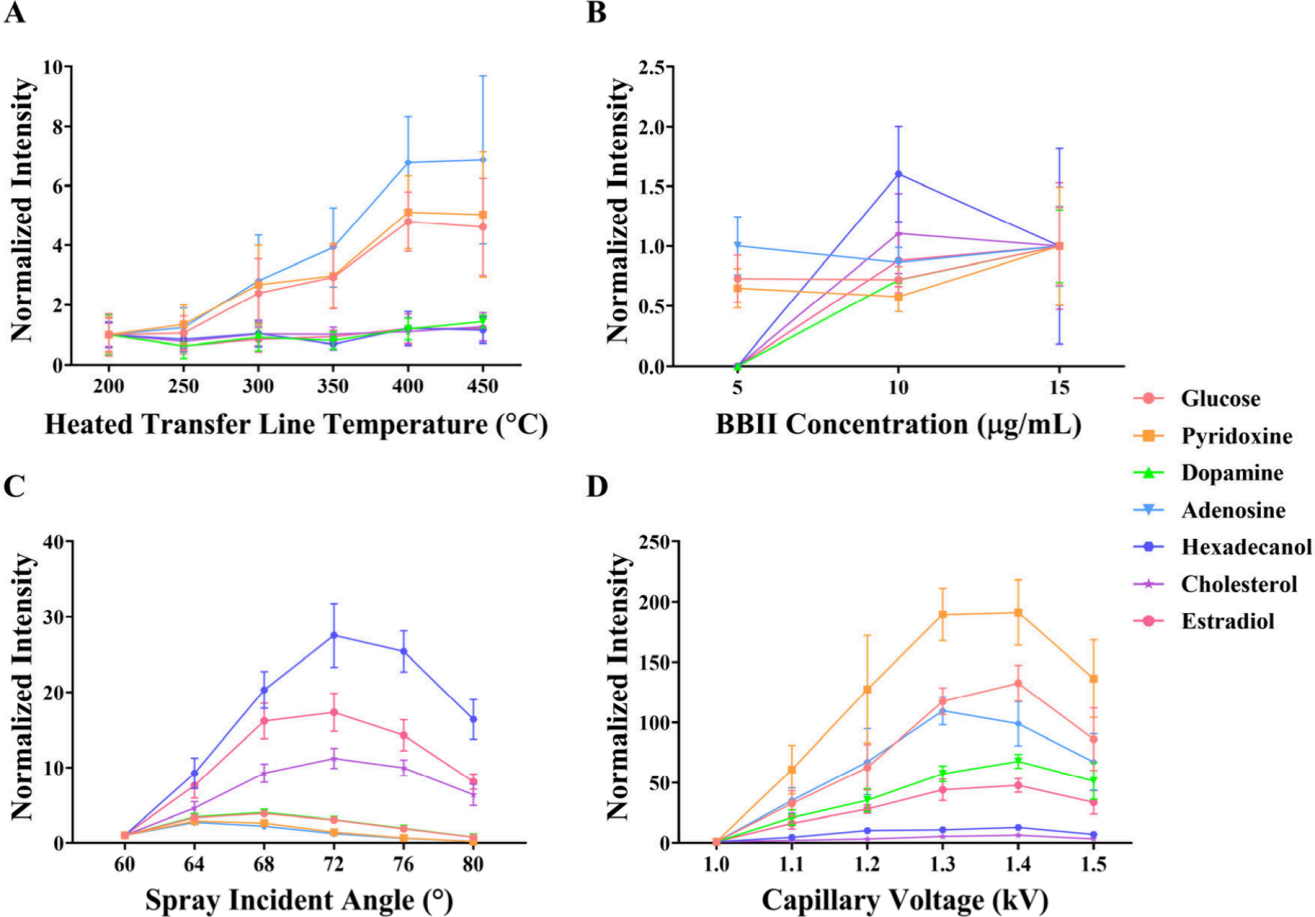
Optimization of critical operational parameters for BBII-DESI analysis. (A) Effect of heated transfer line temperature on signal intensity for hydroxyl metabolites, evaluated across a temperature range of 200–450 °C. (B) Influence of BBII reagent concentration in the spray solvent on derivatization efficiency and signal enhancement, tested at concentrations of 5, 10, and 15 μg/mL. (C) Impact of spray incident angle on ionization efficiency and metabolite detection, optimized between 60° and 80°. (D) Effect of capillary voltage on signal intensity and derivatization performance, evaluated from 1.0 to 1.5 kV.

To optimize the derivatization, BBII concentrations of 5, 10, and 15 μg/mL were evaluated (Figure 3B). At 5 μg/mL, the reaction was clearly incomplete, as dopamine, hexadecanol, cholesterol, and estradiol failed to produce detectable signals. While signals for glucose, pyridoxine, and adenosine were present, the signals for hexadecanol, cholesterol, and estradiol enhanced sharply at 10 μg/mL. Conversely, increasing the concentration to 15 μg/mL resulted in signal suppression for several key analytes and introduced significant signal variability for all compounds. This instability is likely attributed to excess BBII causing either ion suppression of the derivatives or accumulation at the MS inlet, consequently interfering with derivative detection. Therefore, 10 μg/mL was selected as the optimal concentration, providing the best compromise of signal intensity and stability.

Geometric parameters, including spray incident angles, sprayer-to-surface height, and sprayer-to-inlet distance were systematically optimized to achieve optimal derivatization and ionization efficiency. As illustrated in Figure 3C, modulation of the incident angle from 60° to 80° revealed a distinct bifurcated response pattern that was contingent upon the physicochemical properties of the target analytes. Hydrophobic metabolites, specifically hexadecanol, cholesterol, and estradiol, exhibited a characteristic bell-shaped response curve with maximum normalized intensities achieved at 72° followed by a marked decline at 80°. This behavior suggests that the optimal incident angle for hydrophobic compounds facilitates efficient droplet impaction and subsequent desorption/ionization processes. Conversely, hydrophilic metabolites, including glucose, pyridoxine, dopamine, and adenosine, demonstrated a contrasting response profile. These compounds reached maximum normalized intensities at 64° and subsequently exhibited a monotonic decline from 68° to 80°. This differential response likely reflects the distinct solvation and ionization mechanisms governing hydrophilic versus hydrophobic analytes during the spray-based derivatization process. Considering the comprehensive analytical performance across all metabolite classes, an incident angle of 72° was selected as the optimal compromise, providing balanced sensitivity enhancement for both hydrophobic and hydrophilic compounds while maintaining acceptable signal-to-noise ratios for quantitative analysis. The sprayer-to-surface height and sprayer-to-inlet distance significantly influenced derivative signal detection. As demonstrated in Figure S4A and Figure S4B, signal abundance decreased progressively with increasing sprayer-to-surface height. When the height was elevated to 2 mm, derivative signals became undetectable under these conditions. Similarly, the sprayer-to-inlet distance exhibited an analogous pattern, where increased distance adversely affected derivative detection. Therefore, the optimal geometric parameters were determined as sprayer-to-surface height of 0.5 mm and sprayer-to-inlet distance of 1 mm.

For DESI-MS applications, capillary voltage serves as a critical parameter governing charged droplet generation, which directly influences analyte extraction and desorption efficiency from the sample surface. The effect of capillary voltage modulation within the range of 1.0 to 1.5 kV was systematically investigated across all target metabolites. As illustrated in Figure 3D, all metabolites demonstrated a progressive increase in signal abundance from 1.0 to 1.4 kV, followed by a sharp decline at 1.5 kV, confirming the optimal operating voltage at 1.4 kV. This voltage-dependent behavior can be attributed to the competing effects of enhanced ionization efficiency at moderate voltages versus the generation of excessive background noise and potential analyte fragmentation at excessive voltages, which collectively compromise analytical sensitivity and spectral quality. Pyridoxine demonstrated the most pronounced voltage-dependent response, achieving maximum normalized intensity of approximately 190-fold enhancement at 1.4 kV. Glucose and adenosine exhibited moderate responses with peak intensities of approximately 135 and 100-fold enhancement, respectively. The remaining metabolites (dopamine, hexadecanol, cholesterol, and estradiol) showed more modest voltage dependencies, with maximum enhancements ranging from 10 to 65-fold at the optimal voltage. Based on the comprehensive performance evaluation, a capillary voltage of 1.4 kV was selected as the optimal operating parameter.

The effect of nebulizing gas pressure on signal intensity was systematically investigated across a range of 0.03 to 0.15 MPa (Figure S4C). The results demonstrated that signal abundance initially increased with gas pressure, reaching maximum intensity at 0.06 MPa. Beyond this optimal point, further increases in gas pressure resulted in a progressive decline in signal response. Consequently, 0.06 MPa was chosen as the optimal nebulizing gas pressure. It is noteworthy that the boronic acid moiety of BBII may interact with methanol, generating an interfering species which might compete with hydroxyl metabolites and adversely affect the yield of target hydroxyl metabolite derivatives. In our BBII-DESI platform, we confirmed this interaction and detected the interfering species [BBII+MeOH-H_2_O]^+^ (Figure S5). Consequently, methanol, despite being a commonly employed solvent for DESI-MS, was not selected as the solvent base for this application. Optimal performance was achieved using 10 μg/mL BBII in ACN/water (75:25, v/v) at a flow rate of 3 μL/min, a solvent system that provides favorable solubility for both BBII and the target metabolites while facilitating efficient ionization. A spray incident angle of 72°, nitrogen gas pressure of 0.06 MPa, and capillary voltage of 1.4 kV were identified as optimal parameters for maintaining stable spray formation and efficient analyte desorption/ionization. A transfer line temperature of 400 °C was selected based on superior derivatization performance, particularly for hydrophilic metabolites. Under these optimized conditions, various hydroxyl-containing standards exhibited significantly enhanced signal intensities and consistent derivatization efficiency, demonstrating the robustness and reliability of the BBII-DESI method across diverse analyte classes.

### Reaction Efficiency and Signal Enhancement of Hydroxyl Metabolites by BBII-DESI

To comprehensively evaluate the analytical performance of the BBII-DESI methodology, we conducted a systematic assessment of ionization efficiency and derivatization effectiveness using a representative panel of hydroxyl-containing metabolite standards under optimized conditions. The integration of BBII into the DESI spray solvent enabled rapid *in situ* derivatization upon droplet impact with the sample surface, yielding substantial signal enhancements across all tested analytes.

Comparative analysis of signal intensities between underivatized standards analyzed by conventional DESI-MS and BBII-derivatized analytes revealed consistent and statistically significant improvements ranging from 1.8- to 17.2-fold (Figure 4). Pyridoxine demonstrated the most pronounced enhancement (17.2-fold increase), followed by dopamine (5.1-fold), cholesterol (3.4-fold), and adenosine (1.8-fold). The relatively modest enhancement observed for adenosine can be attributed to its inherently high ionization efficiency in the underivatized state, consistent with our previous investigation.^31^ Most notably, metabolites that were below the detection threshold in positive ion mode DESI-MS, including glucose, hexadecanol, and estradiol, became readily detectable following BBII derivatization. The substantial signal enhancements provide compelling evidence for the analytical utility of BBII-DESI in biological applications. The observed improvements in detection sensitivity, particularly for previous inaccessible metabolites, significantly expand BBII-DESI’s applicability for trace-level analysis in complex biological matrices.

**Figure 4:**
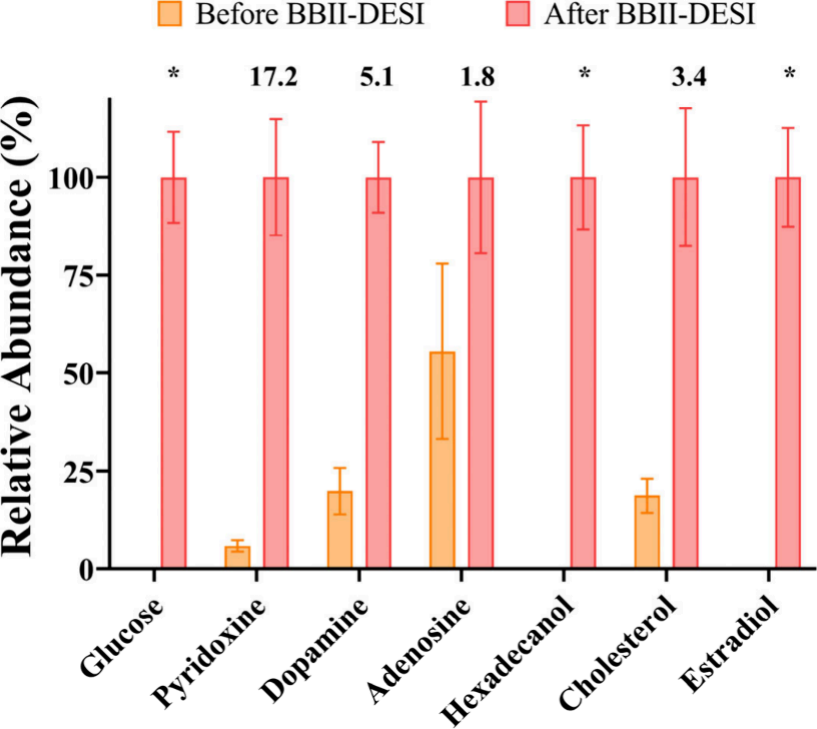
Relative abundance of hydroxyl metabolite standards before and after BBII-DESI derivatization with calculated signal enhancement factors. Asterisks indicate metabolites that were undetectable in positive ion mode before derivatization and became detectable after BBII-DESI.

In conclusion, these findings substantiate the efficacy of BBII-assisted derivatization in facilitating enhanced ionization of hydroxyl-containing metabolites under ambient DESI conditions, yielding considerable improvements in detection sensitivity without requiring offline sample preparation protocols. For quantitative applications in biological samples, we recommend the incorporation of isotopically labeled internal standards to ensure analytical accuracy and precision. The demonstrated signal enhancements and expanded detection capabilities underscore the significant potential of BBII-DESI for comprehensive hydroxyl metabolite profiling in complex biological systems.

### *In Situ* BBII-DESI for Mouse Brain Tissue Samples

Hydroxyl metabolites serve critical functions in brain physiology, including cholesterol transport regulation, neurotransmitter signaling modulation, and serving as indicators of pathological states or drug metabolism.^15,32,33^ MSI provides a powerful platform for visualizing and quantifying these metabolites across distinct brain regions, thereby revealing spatial heterogeneity in their distribution and abundance.^12,26^ However, the detection of hydroxyl metabolites presents significant analytical challenges due to their inherently low ionization efficiency, which can compromise sensitivity in conventional MSI approaches.^34–36^ Having demonstrated the efficacy of the BBII-DESI method for enhancing detection sensitivity of hydroxyl metabolite standards in the aforementioned experiments, we proceeded to evaluate its potential within the complex biological matrix of mouse brain tissue. This application represents a crucial validation step, as the biological environment presents additional analytical challenges including heterogeneous tissue architecture, variable metabolite concentrations, and complex background interferences that can confound detection efforts. Our BBII-DESI methodology enabled real-time, *in situ* derivatization and detection of endogenous hydroxyl metabolites directly from the tissue surface, transforming analytically challenging compounds into readily ionizable derivatives while preserving their spatial distribution information. Systematic analysis revealed numerous features exhibiting the distinctive boron isotopic pattern (Table S1).

Figure 5 presents compelling evidence of the method’s capabilities through representative mass spectrometry images of key hydroxyl metabolites, including glucose and cholesterol, acquired before and after BBII-DESI treatment. The contrast is striking, while these metabolites were essentially undetectable or exhibited extremely weak signals in conventional underivatized analysis, BBII-DESI treatment revealed their abundance and distinct spatial distributions. Glucose, a fundamental brain energy substrate, exhibited well-defined regional localization patterns that correlated with known areas of high metabolic activity;^37^ and cholesterol, essential for membrane integrity and myelin formation, showed characteristic enrichment in white matter regions.^36,38^ The method revealed metabolic microenvironments and regional specialization that provide new insights into brain organization and function. Furthermore, the characteristic isotopic pattern of boron provided an invaluable molecular signature that served as a selective screening tool for identifying potential derivative signals within complex mass spectra, thereby increasing confidence in metabolite assignments while reducing false discovery rates.

**Figure 5:**
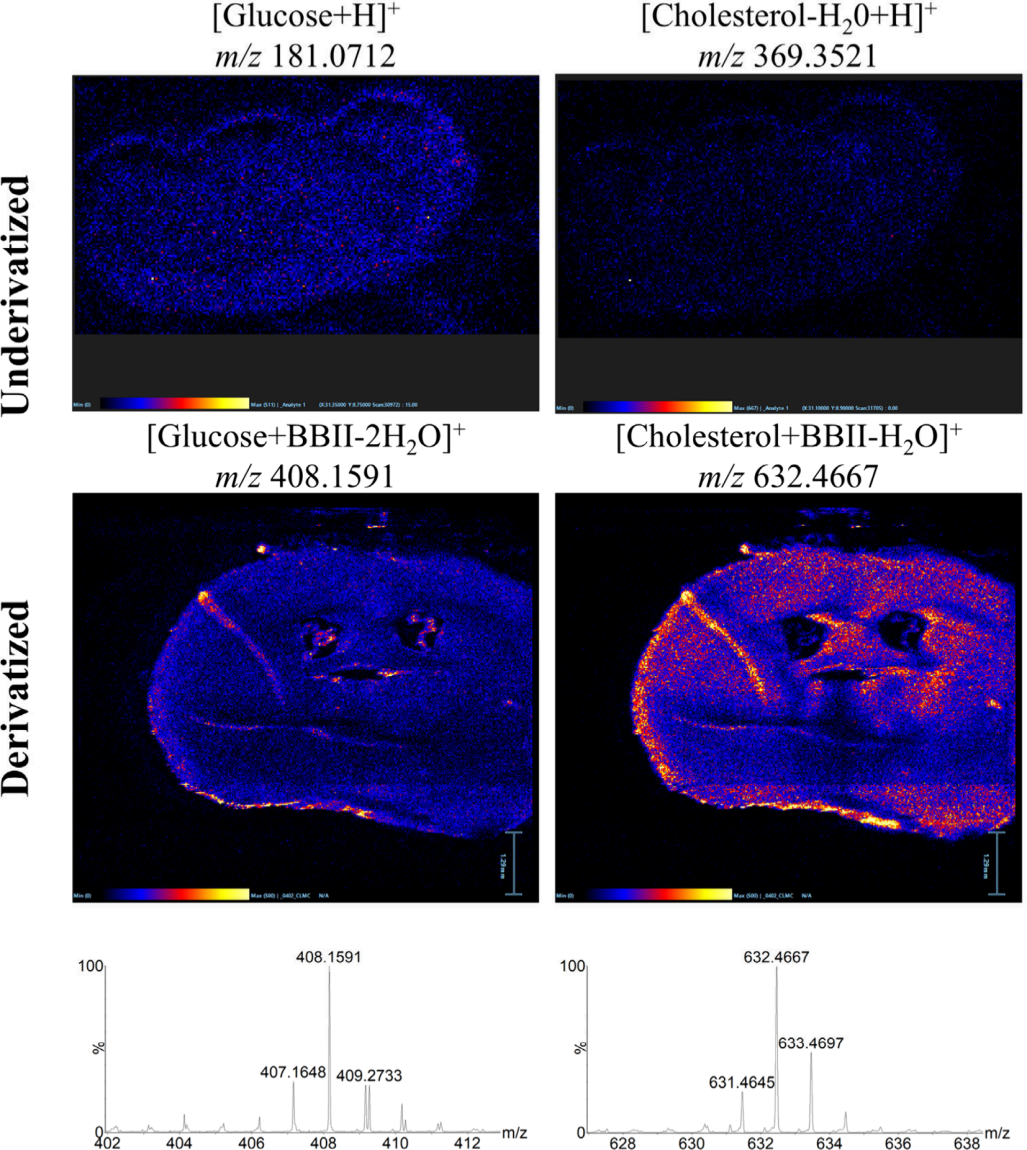
Mass spectrometry images of representative hydroxyl metabolites (glucose and cholesterol) in mouse brain tissue section before and after BBII-DESI derivatization. Images were acquired in positive ion mode with 25 μm spatial resolution. The derivatized metabolites showed characteristic boron isotopic patterns, enabling rapid screening and selective identification.

The analytical capacity of BBII-DESI extended well beyond the detection of derivatized hydroxyl metabolites, enabling a comprehensive characterization of the metabolic landscape of brain tissue. In addition to enhanced signals from BBII-derivatized compounds, numerous endogenous metabolites and lipids were detected, with 37 features tentatively identified from mouse brain tissue (Figure S6, Table S2). These findings demonstrate that BBII-DESI preserves broad metabolomic coverage while simultaneously accessing hydroxyl metabolites that are typically inaccessible by conventional DESI-MS. Among the derivatized metabolites (Figure 6), we confidently identified glycerol (*m*/*z* 320.1475), a central component in membrane lipid metabolism;^39^ uridine (*m*/*z* 472.1665), an essential nucleoside involved in RNA synthesis and neuroplasticity;^40^ and monoacylglycerols MG (16:0) (*m*/*z* 558.3709), MG (18:1) (*m*/*z* 584.3882), and MG (18:2) (*m*/*z* 600.3884), which serve as important signaling molecules and metabolic intermediates.^41^ Notably, inosine was observed at *m*/*z* 496.1761 and 514.1929, corresponding to [inosine+BBII-2H_2_O]^+^ and [inosine+BBII-H_2_O]^+^, respectively. This suggests that inosine exhibits particularly high reactivity toward BBII, resulting in an intense primary derivative at *m*/*z* 496.1761 and a minor secondary derivative at *m*/*z* 514.1929.

**Figure 6:**
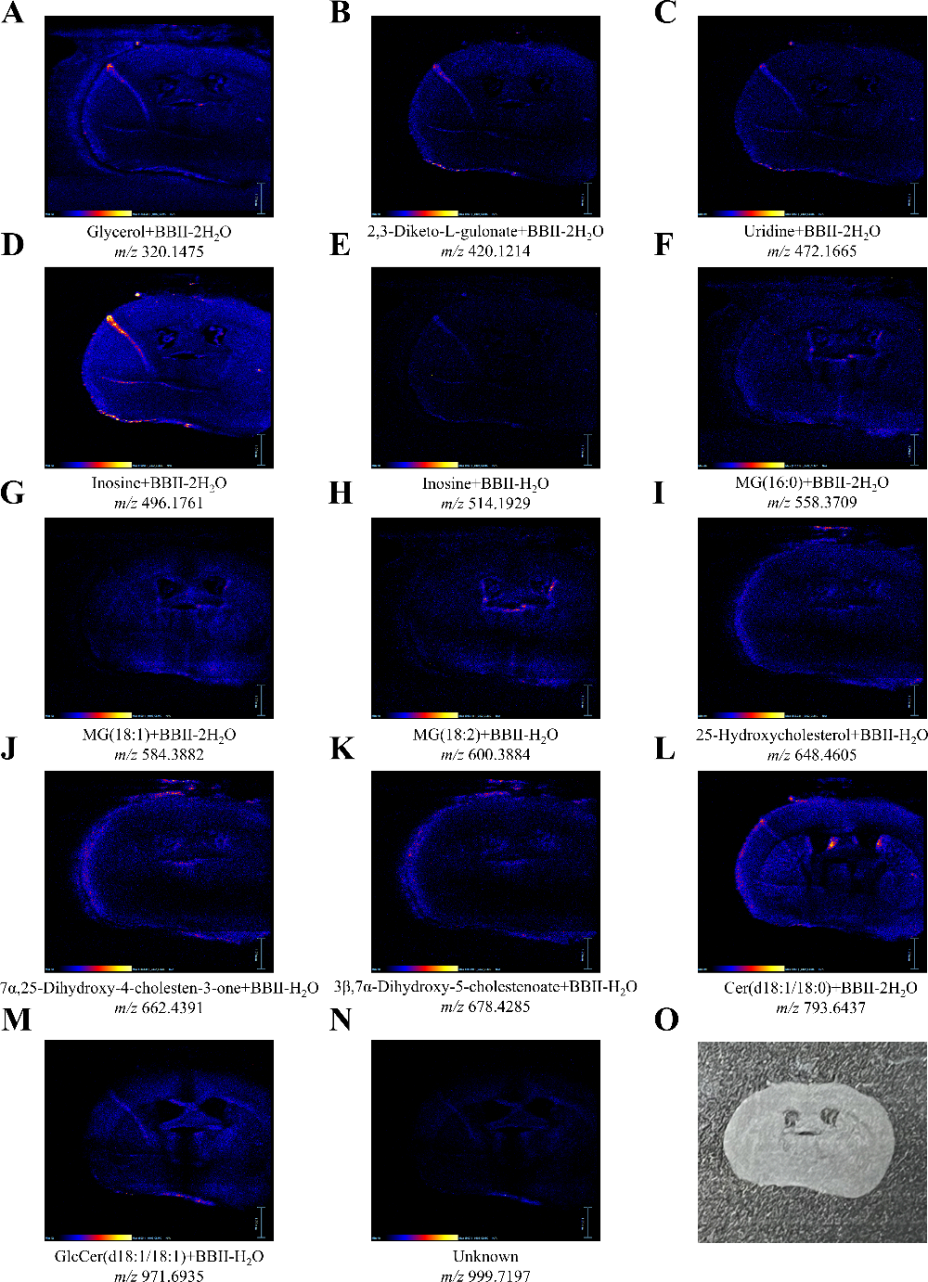
Mass spectrometry images show spatial distributions of BBII-derivatized hydroxyl metabolites in mouse brain tissue section. Fourteen representative features are displayed with their corresponding *m*/*z* values, including tentatively identified compounds based on mass deconvolution (subtracting BBII reagent mass and adding one or two water molecules) followed by accurate mass matching of the parent metabolites: (A) glycerol, (B) 2,3-diketo-L-gulonate, (C) uridine, (D) inosine, (E) inosine, (F) monoacylglycerols MG (16:0), (G) MG (18:1), (H) MG (18:2), (I) 25-hydroxycholesterol, (J) 7α,25-dihydroxy-4-cholesten-3-one, (K) 3β,7α-dihydroxy-5-cholestenoate, (L) Cer­(d18:1/18:0), (M) GlcCer­(d18:1/18:1), (N) unidentified feature at *m*/*z* 999.7197, and (O) optical image of mouse brain tissue section. All features exhibited characteristic boron isotopic patterns confirming successful derivatization.

Remarkably, 25-hydroxycholesterol, 7α,25-dihydroxy-4-cholesten-3-one, and 3β,7α-dihydroxy-5-cholestenoate were also detected. These metabolites represent key intermediates in the neuronal cholesterol oxidation cascade and the primary bile acid biosynthesis pathway. In the central nervous system, cholesterol serves not only as a major structural component of neuronal membranes and myelin but also as the metabolic precursor for oxysterols such as 25-hydroxycholesterol, which functions as a potent regulator of cholesterol homeostasis, lipid metabolism, and immune signaling within the brain.^42,43^ The detection of 7α,25-dihydroxy-4-cholesten-3-one and 3β,7α-dihydroxy-5-cholestenoate is particularly significant, as these metabolites lie downstream of cholesterol oxidation and participate in the enzymatic route linking cholesterol turnover to bile acid synthesis. This pathway has gained increasing attention in neurobiology for its emerging role in neurosteroid biosynthesis, glial cell signaling, and neuroprotective regulation of cholesterol clearance.^44–46^ These findings demonstrate that BBII-DESI can reveal functionally relevant sterol intermediates that are often overlooked due to their low abundance and poor ionization efficiency.

By enabling *in situ* visualization and annotation of a wide spectrum of hydroxyl metabolites, the BBII-DESI platform provides a powerful analytical tool for revealing previously inaccessible dimensions of brain chemistry. Beyond sterol intermediates, BBII-DESI successfully detected diverse hydroxyl metabolites such as glucose, monoacylglycerols, and inosine. This expanded chemical coverage demonstrates the method’s capability to complement conventional DESI-MS by capturing low-abundance or weakly ionizing hydroxyl metabolites. Collectively, these findings establish BBII-DESI as a sensitive and label-free molecular imaging approach that enhances metabolome coverage, supports spatially resolved metabolomics, and advances our understanding of metabolic dynamics in health, disease, and neurochemical regulation.

## Conclusions

In summary, we successfully developed the BBII-DESI approach, enabling efficient *in situ* derivatization directly on sample surfaces. By incorporating BBII into the DESI spray solvent, we overcome the inherent ionization limitations of polar, neutral hydroxyl compounds, leading to substantial signal enhancements (1.8- to 17.2-fold) and making previously challenging analytes such as glucose, fatty alcohols, and steroids readily detectable by mass spectrometry. The utility of BBII-DESI was further demonstrated through its application to mouse brain tissue sections, which provided high-sensitivity molecular imaging while revealing distinct spatial distributions of hydroxyl metabolites like glucose and cholesterol with improved resolution compared to nonderivatized DESI-MSI. Unlike conventional approaches requiring labor-intensive extraction procedures, extensive sample preparation steps, or off-line derivatization protocols that risk spatial information loss, our method achieves instantaneous chemical modification and ionization enhancement while maintaining the integrity of tissue morphology and metabolite localization. The unique boron isotope signature provided confident identification of derivatized analytes, thereby enhancing analytical specificity and expanding metabolome coverage beyond conventional methods. These findings establish BBII-DESI as a significant advancement in ambient mass spectrometry, broadening the scope of metabolite analysis and opening new opportunities for high-throughput screening, tissue-based diagnostics, and detailed metabolic investigation in biological systems. Future efforts will focus on expanding its application to a wider array of biological samples and exploring its potential for quantitative metabolite imaging and novel biomarker discovery.

## Supplementary Material



## Notes

Mass spectra of standard hydroxyl compounds comparing Method 1 and Method 2 (Figure S1); chemical structures of BBII and derivatized hydroxyl metabolite standards (Figure S2); mass spectra of minor derivatives of adenosine and pyridoxine, and BBII-derivatized hydroquinone (Figure S3); optimization of additional DESI experimental parameters (Figure S4); mass spectrum showing BBII reaction with MeOH (Figure S5); mass spectrometry images of underivatized metabolites in mouse brain tissue (Figure S6); BBII-derivatized metabolites detected in mouse brain tissue (Table S1); underivatized metabolites detected in mouse brain tissue (Table S2) (PDF)
