# Supplementary material for: Enhancing Hydroxyl Metabolite Analysis through In Situ Derivatization-Enabled Desorption Electrospray Ionization-Mass Spectrometry
Source: Anal Chem. 2025 Nov 14;97(48):26570–9. doi: 10.1021/acs.analchem.5c04858 (PMC12874211; doi:10.1021/acs.analchem.5c04858)
Supplement: Supplementary file 1 [file ac5c04858_si_001.pdf]

Supporting Information

Enhanced Hydroxyl Metabolite Detection by BBII Derivatization Coupled with DESI-MS

Yen-Chu Lin [a,#], Guan-Yuan Chen [b,c,#], Ya-Jin Jheng [b,c], and Hsiao-Wei Liao [a,*]

[a] Department of Pharmacy, College of Pharmaceutical Sciences, National Yang Ming Chiao Tung University, No.155, Sec.2, Linong Street, Taipei 112304, Taiwan.
[b] Forensic and Clinical Toxicology Center, National Taiwan University, No.7, Zhongshan S. Rd., Zhongzheng Dist., Taipei 100225, Taiwan.
[c] Department and Graduate Institute of Forensic Medicine, College of Medicine, National Taiwan University, No.7, Zhongshan S. Rd., Zhongzheng Dist., Taipei 100225, Taiwan.

#These authors contributed equally.

*Corresponding Author.
Address: Department of Pharmacy, National Yang Ming Chiao Tung University, No.155, Sec.2, Linong Street, Taipei 112304, Taiwan
Tel.: +886-2-2826-7927
E-mail: hsiaoweiliao@nycu.edu.tw

Table of Contents

Figure S1 .... S-2
Figure S2 .... S-3
Figure S3 .... S-4
Figure S4 .... S-5
Figure S5 .... S-6
Figure S6 .... S-7
Table S1 .... S-14
Table S2 .... S-15

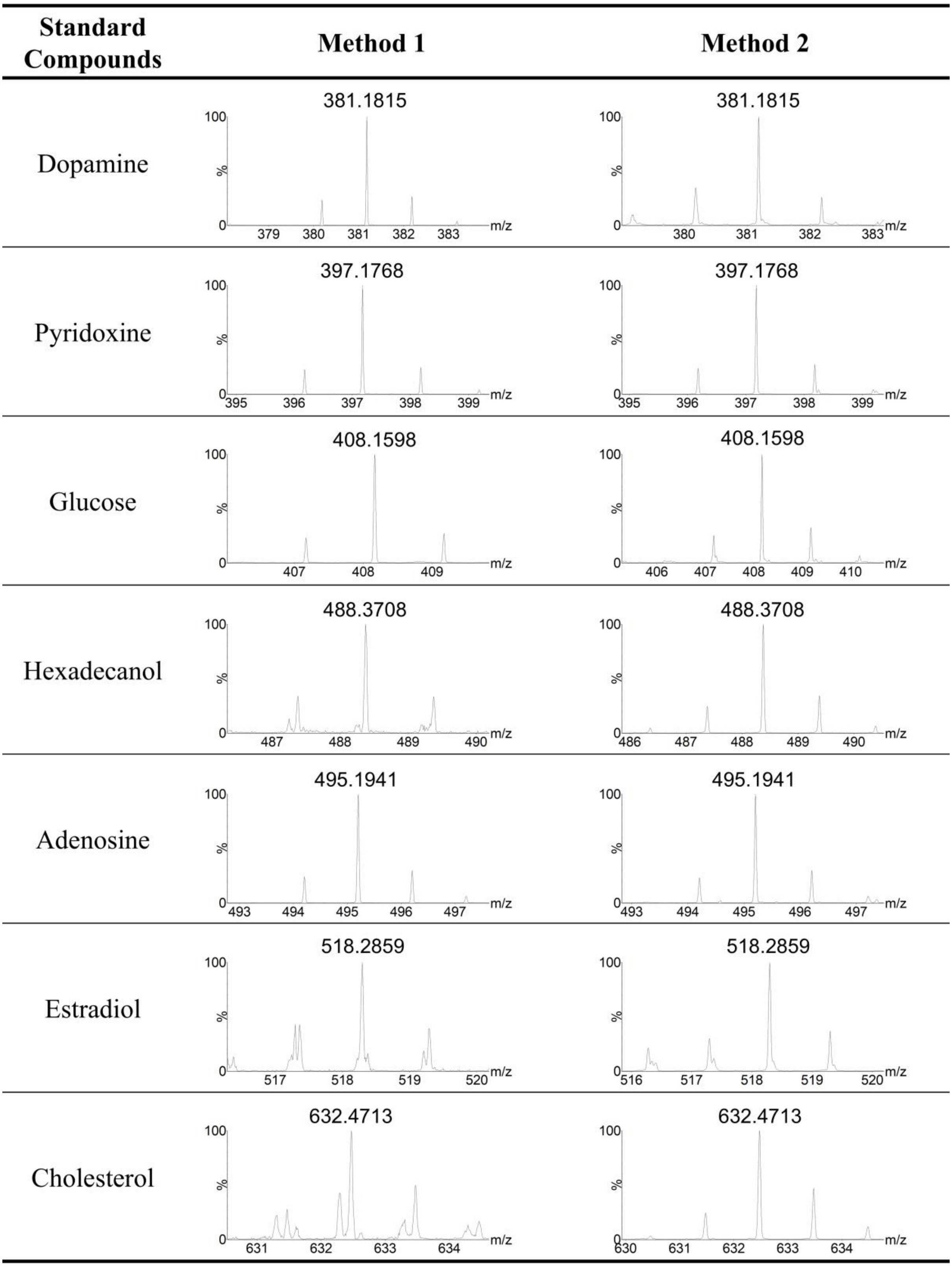

Figure S1. Mass spectra of the standard hydroxyl compound, comparing results obtained using Method 1 (pre-spraying the BBII reagent onto the sample surface prior to DESI ionization) and Method 2 (incorporating the BBII reagent directly into the spray solvent).

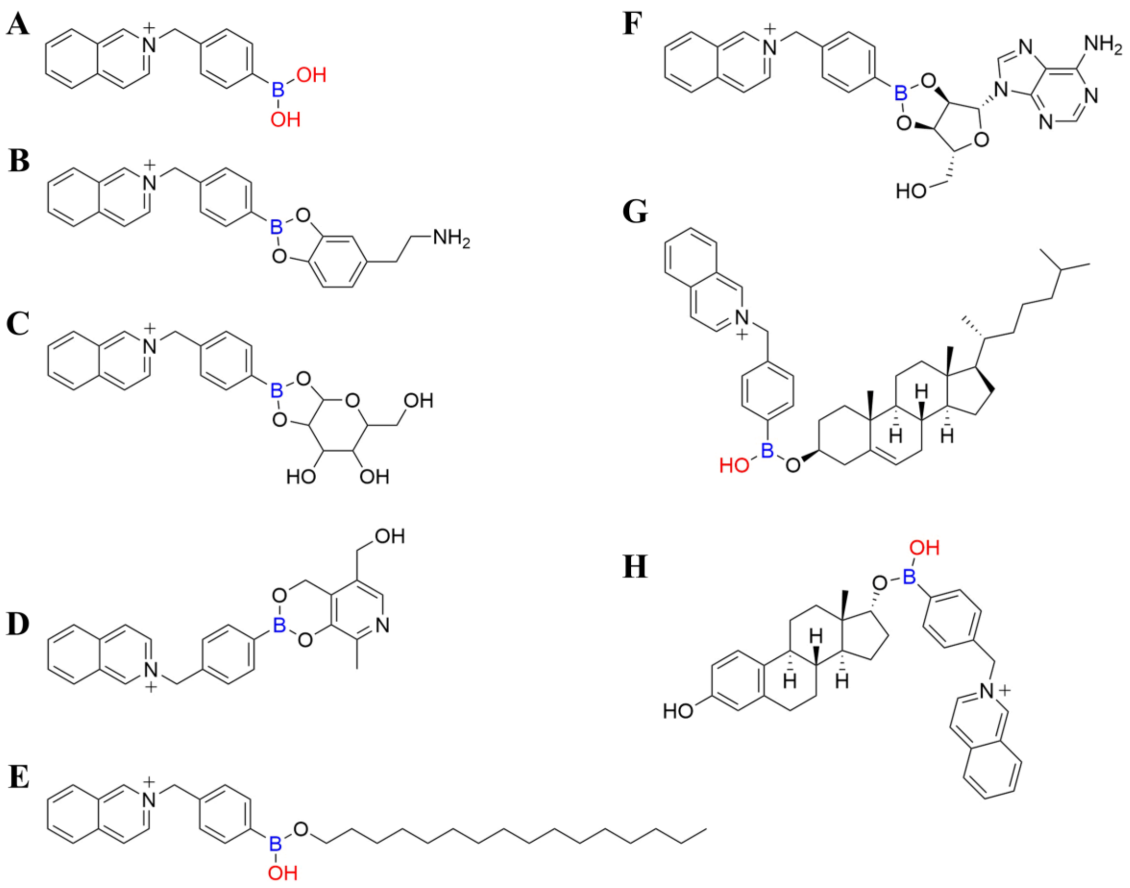

Figure S2. Chemical structures of (A) BBII and derivatized hydroxyl metabolite standards: (B) BBII-dopamine, (C) BBII-glucose, (D) BBII-pyridoxine, (E) BBII-hexadecanol, (F) BBII-adenosine, (G) BBII-cholesterol, and (H) BBII-estradiol.

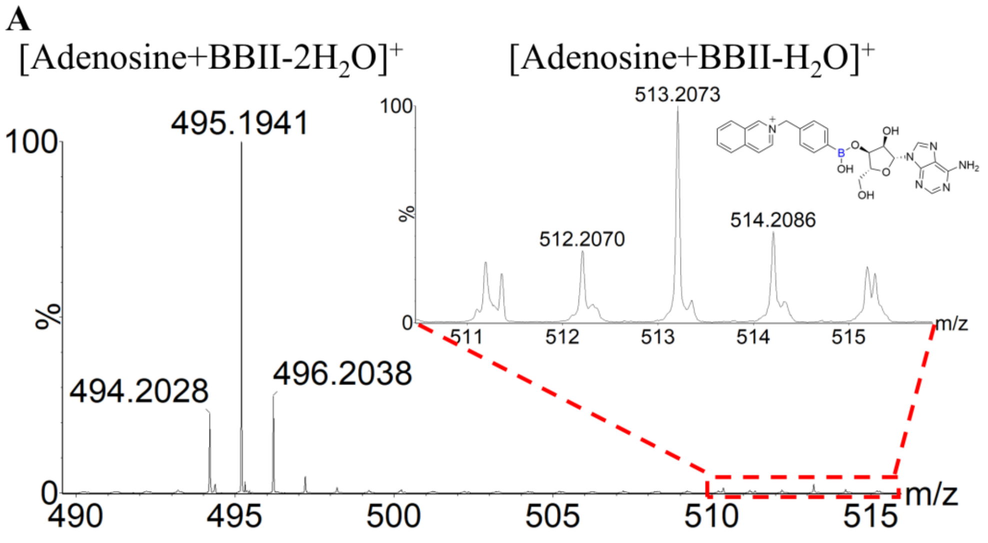

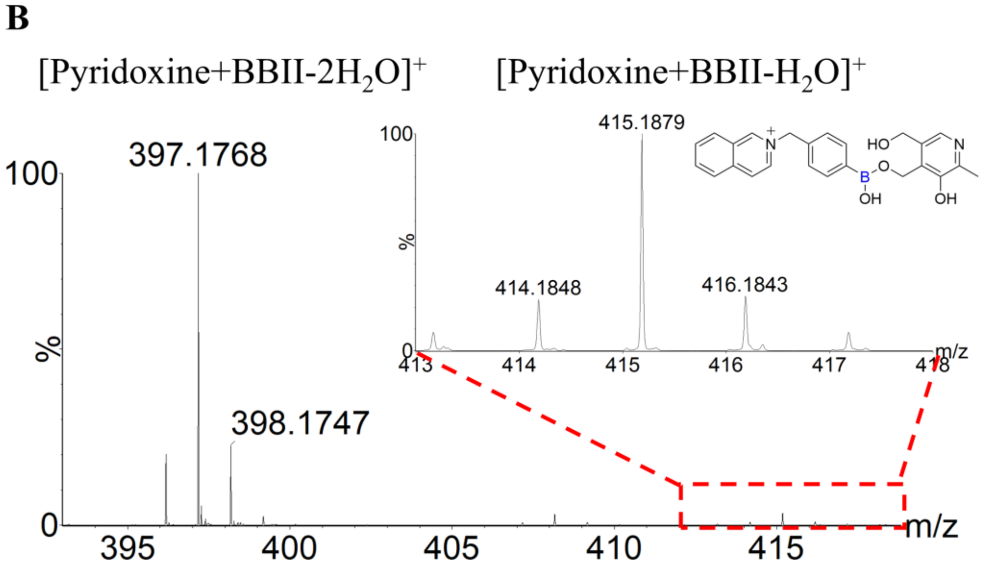

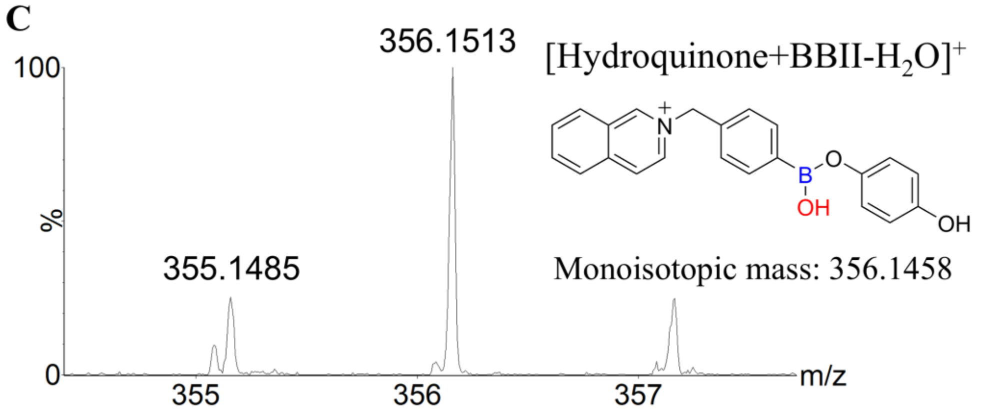

Figure S3. Minor derivatives of (A) adenosine and (B) pyridoxine. The minor derivatives of adenosine (*m/z* 513.2073) and pyridoxine (*m/z* 415.1879) correspond to [adenosine+BBII-H2O]$^+$ and [pyridoxine+BBII-H2O]$^+$, respectively. (C) Mass spectrum of BBII-derivatized hydroquinone (*m/z* 356.1513).

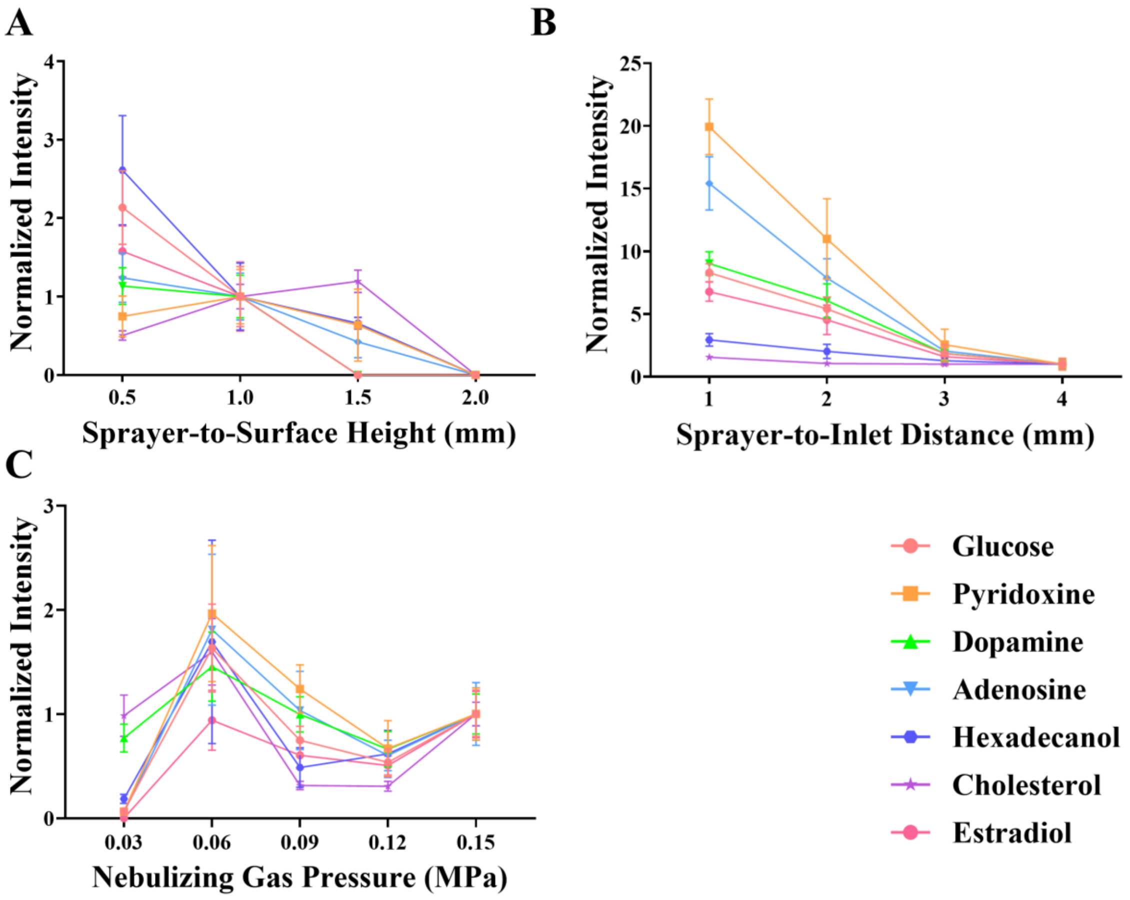

Figure S4. Additional DESI experimental parameters optimization. (A) Sprayer-to-surface height, ranging from 0.5 to 2.0 mm. (B) Sprayer-to-inlet distance, ranging from 1 to 4 mm. (C) Nebulizing gas pressure, ranging from 0.03 to 0.15 MPa.

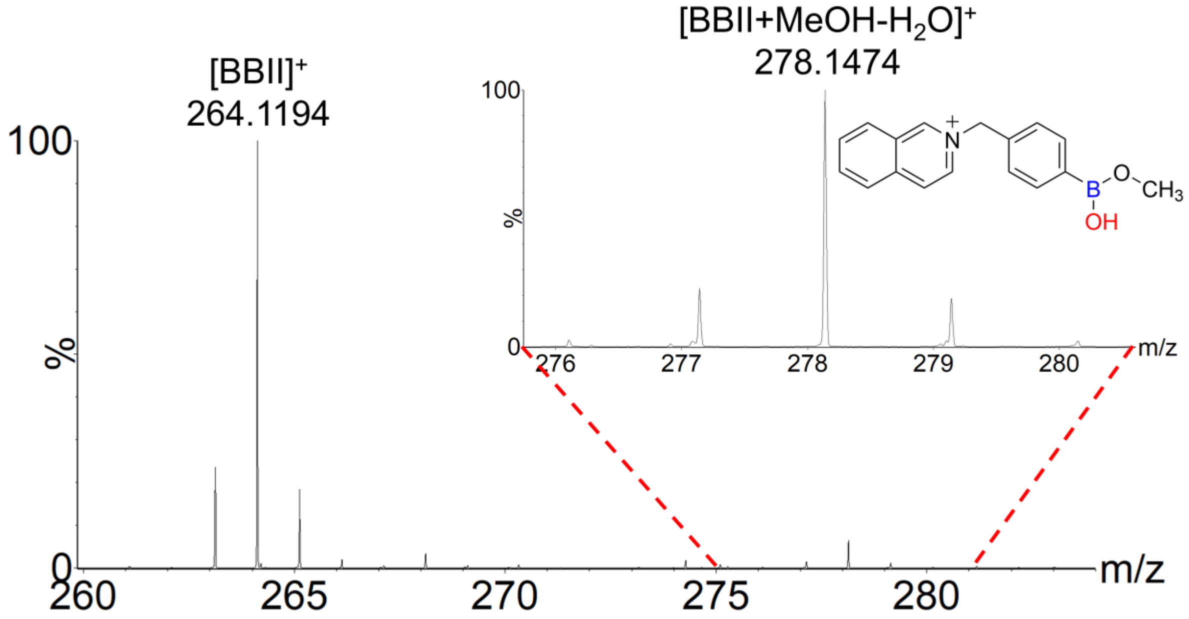

Figure S5. BBII reacts with MeOH to form [BBII+MeOH-H_2O]$^+$ at *m/z* 278.1474.

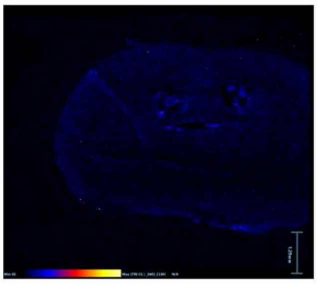
Unknown
m/z 148.1117

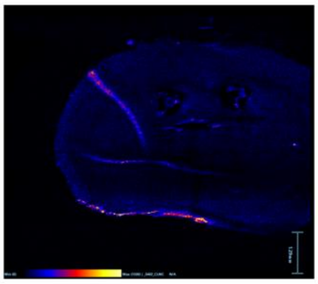
L-Carnitine
m/z 162.1126

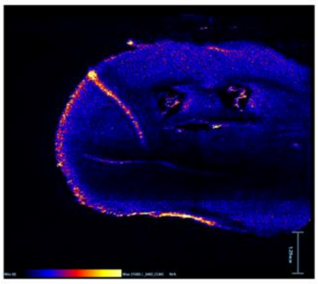
Phosphorylcholine
m/z 184.0740

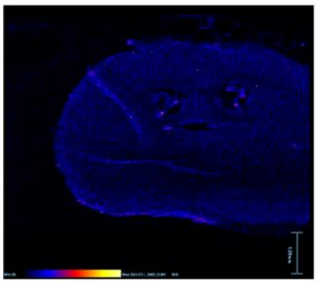
Unknown
m/z 196.8779

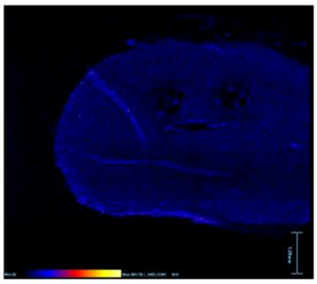
Unknown
m/z 212.8519

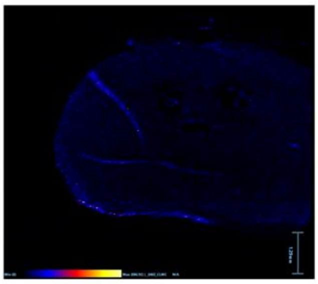
Unknown
m/z 307.0461

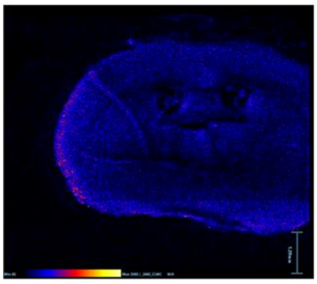
Unknown
m/z 429.2389

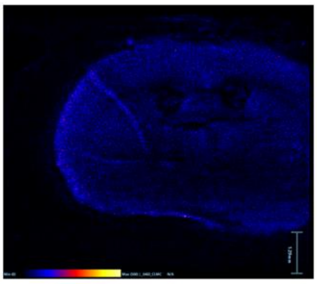
Unknown
m/z 441.192

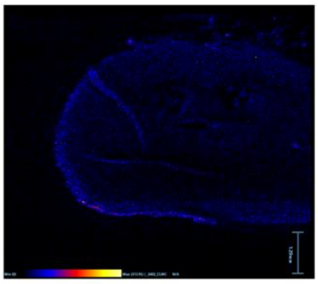
Unknown
m/z 483.0741

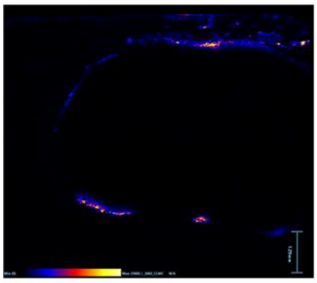
Unknown
m/z 529.253

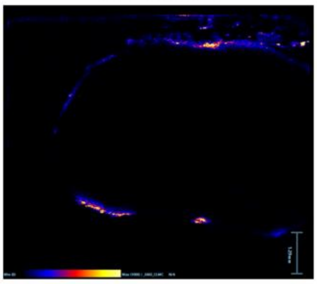
Unknown
m/z 543.9286

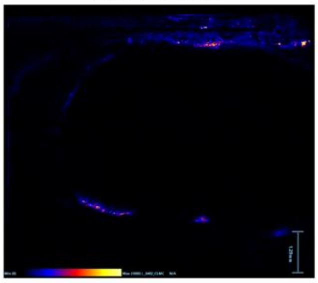
Unknown
m/z 553.2753

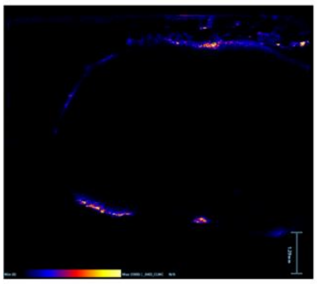

Unknown
m/z 558.6018

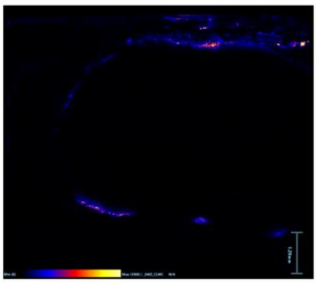

Unknown
m/z 568.2874

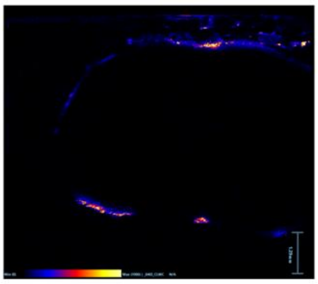

Unknown
m/z 573.2831

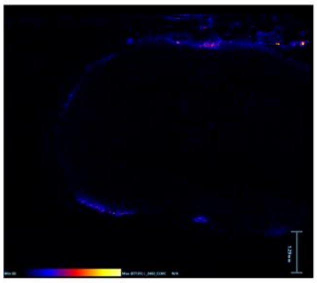

Unknown
m/z 576.2816

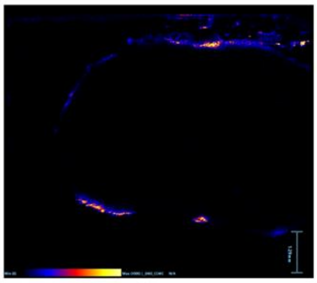

Unknown
m/z 587.9557

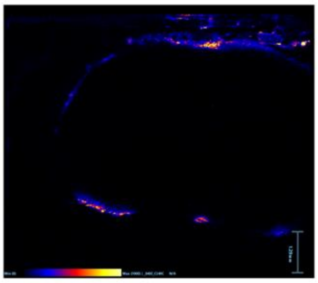

Unknown
m/z 598.2929

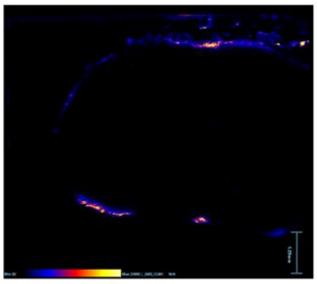

Unknown
m/z 602.6315

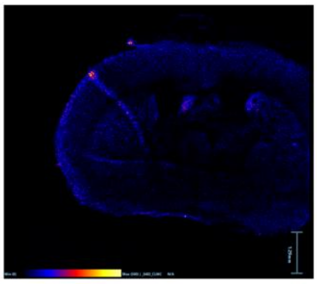

Cer(d18:1/18:0)
m/z 604.5038

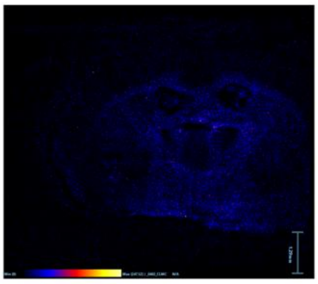

Unknown
m/z 606.3696

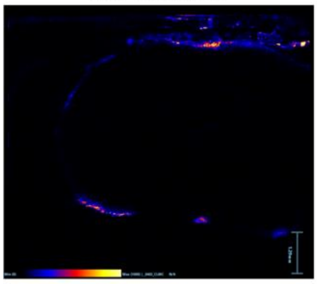

Unknown
m/z 612.3127

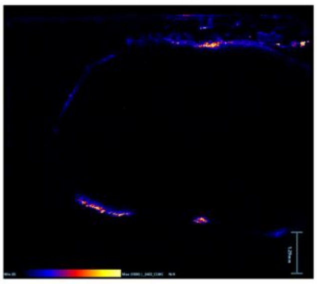

Unknown
m/z 617.3036

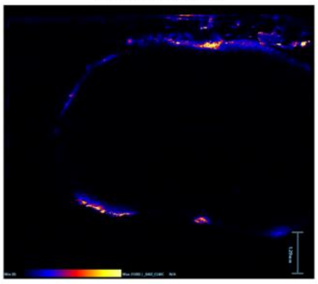

Unknown
m/z 620.3079

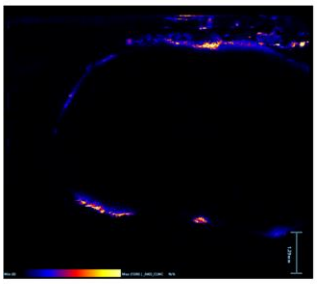

Unknown
m/z 642.3243

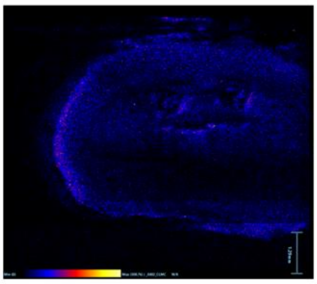

Unknown
m/z 646.4417

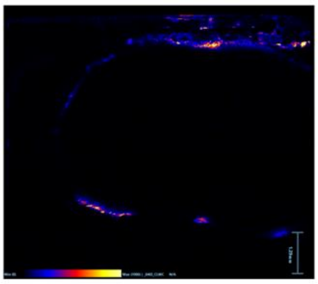

Unknown
m/z 656.3471

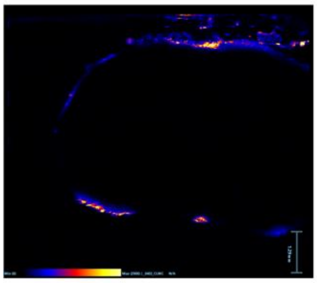

PC(24:6;2OH)
m/z 664.3317

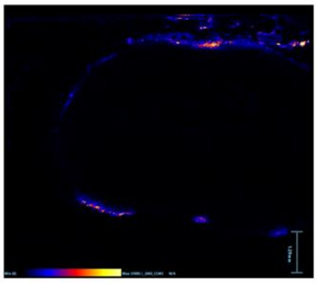

Unknown
m/z 678.3572

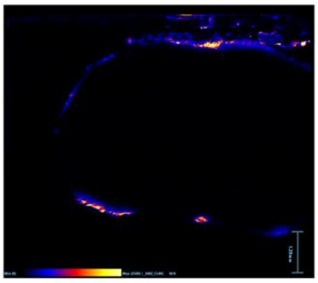

Unknown
m/z 686.3513

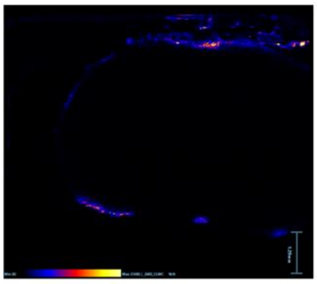

Unknown
m/z 700.3683

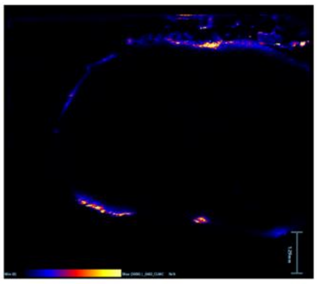

Unknown
m/z 708.3658

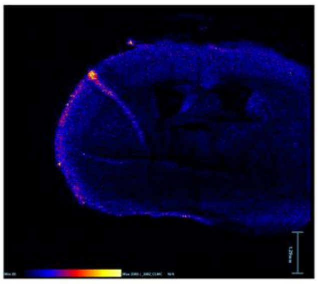

PA(34:1)
m/z 713.4513

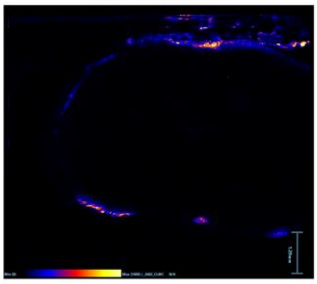

Unknown
m/z 722.3842

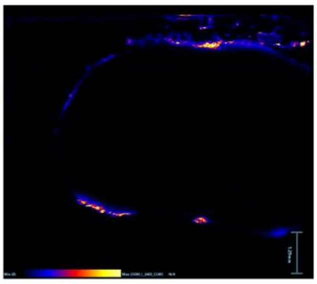

Unknown
m/z 730.3793

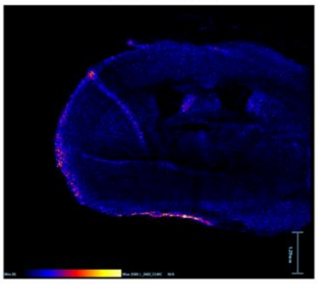

SM(36:1)
m/z 731.6051

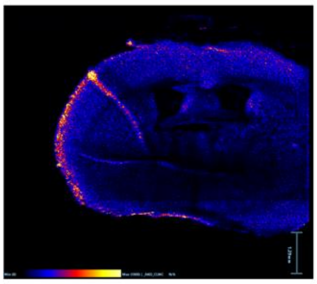
PC(32:0)
m/z 734.5681

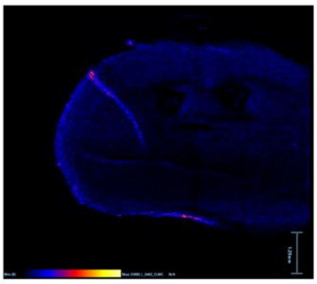
Unknown
m/z 739.4703

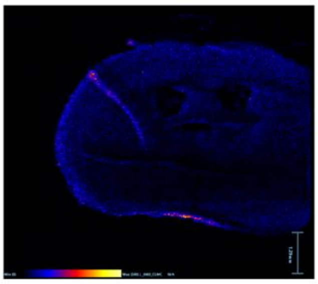
PE(38:7)
m/z 744.4954

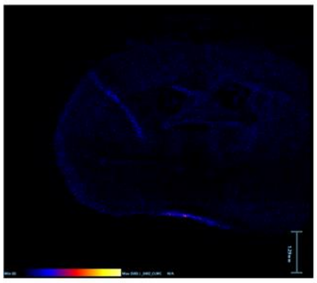
PE(P-38:5)
m/z 750.5543

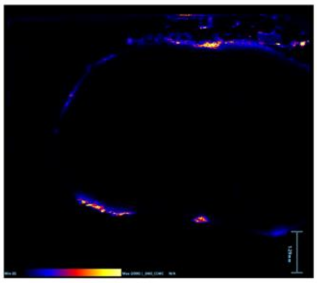
Unknown
m/z 752.3864

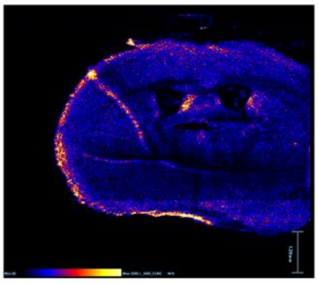
SM(36:1)
m/z 753.5876

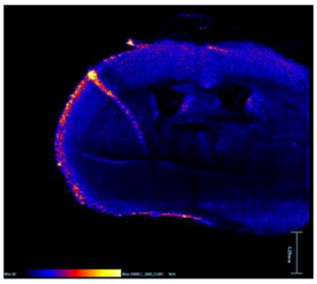
PC(32:0)
m/z 756.5518

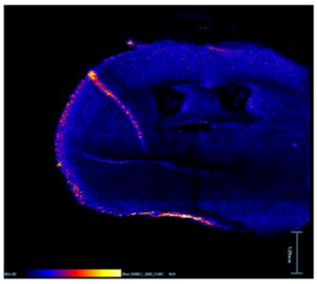
PC(34:1)
m/z 760.5886

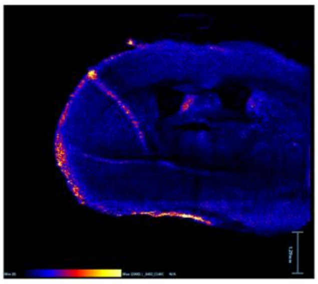
SM(d18:1/18:0)
m/z 769.5615

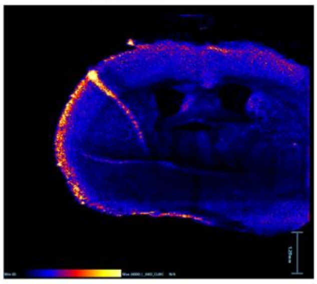
PE(P-38:5)
m/z 772.5243

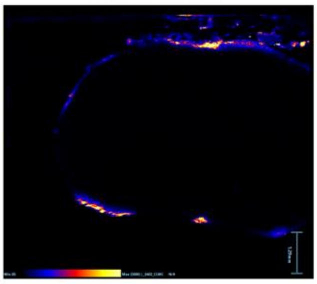
Unknown
m/z 774.4048

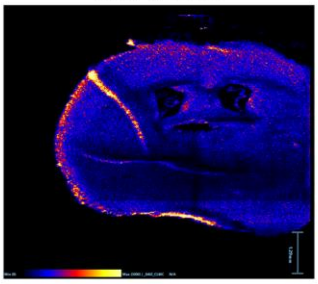
PC(34:1)
m/z 782.5618

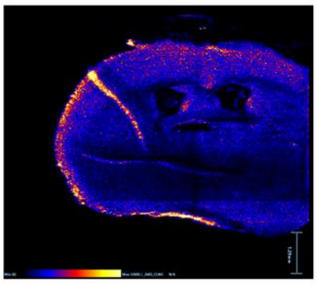

PC(34:0)
m/z 784.5748

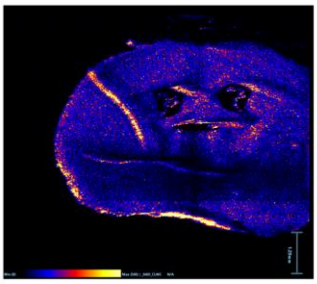

PC(36:1)
m/z 788.6197

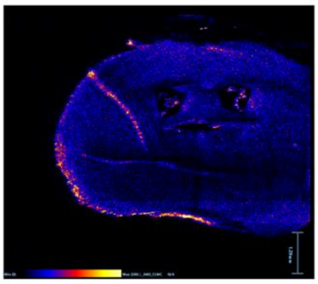

Unknown
m/z 790.5197

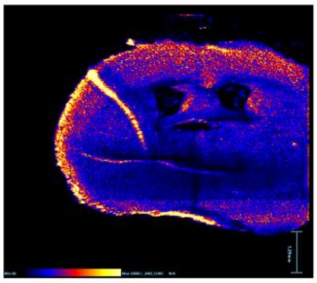

PC(34:1)
m/z 798.5400

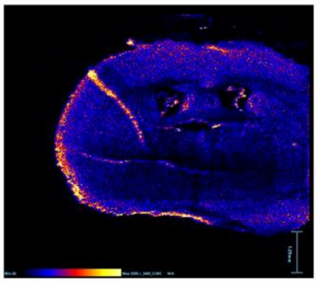

PE(38:4)
m/z 806.5119

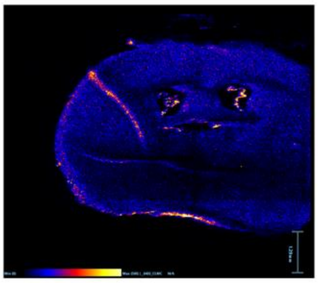

PC(38:5)
m/z 808.5778

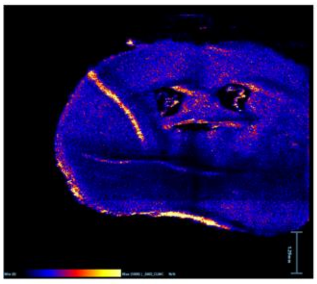

PC(38:4)
m/z 810.6017

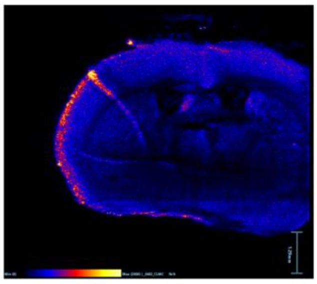

Unknown
m/z 814.5346

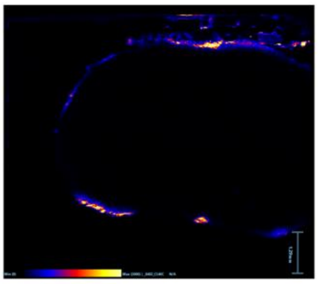

Unknown
m/z 818.4323

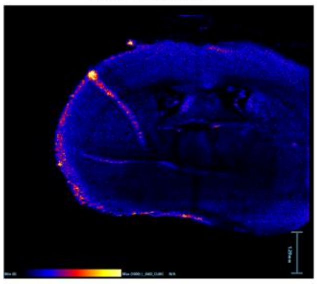

PC(36:4)
m/z 820.5245

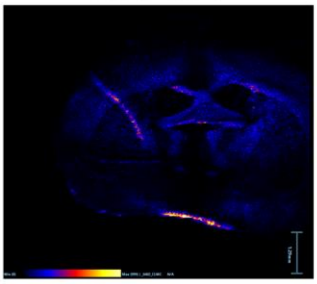

GalCer(d18:1/22:0)
m/z 822.6306

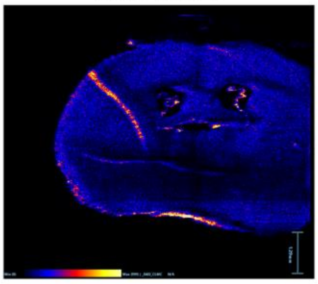

PC(36:2)
m/z 824.5599

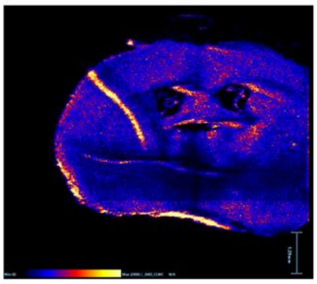

PC(36:1)
m/z 826.5701

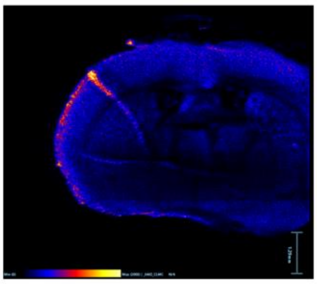

PE(40:6)
m/z 830.5189

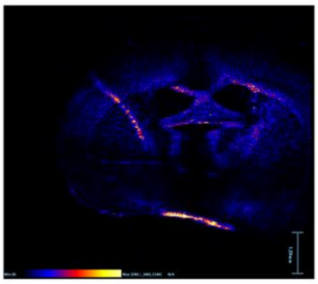

PC(O-38:5)
m/z 832.5701

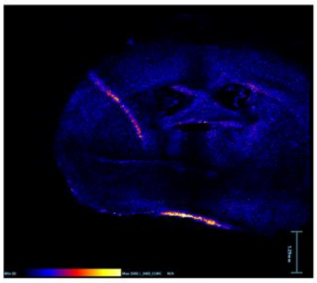

PC(40:5)
m/z 836.6237

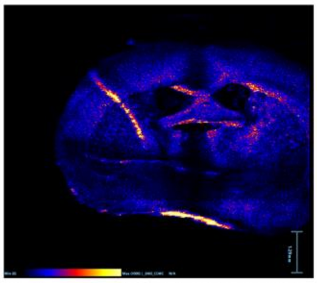

GlcCer(d18:1(8Z)/22:0(2OH[R]))
m/z 838.6259

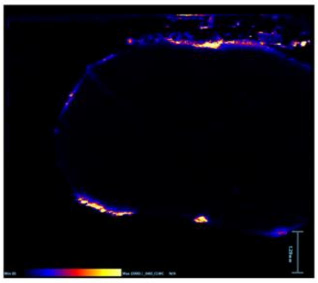

Unknown
m/z 840.4378

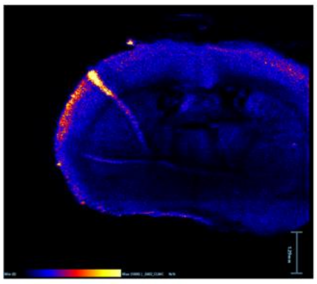

PC(38:6)
m/z 844.5218

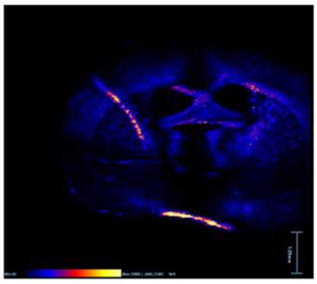

GalCer(d18:1/24:1(15Z))
m/z 848.6385

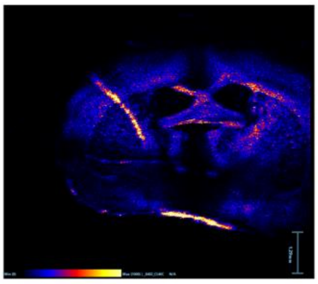

GalCer(d18:1/24:0)
m/z 850.655

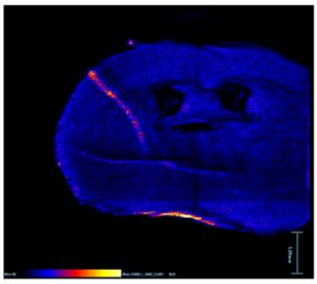

SM(d18:1/26:1(17Z))
m/z 863.6884

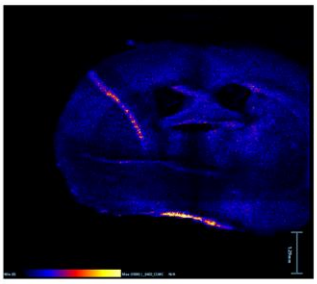

LacCer(d18:0/16:0)
m/z 864.642

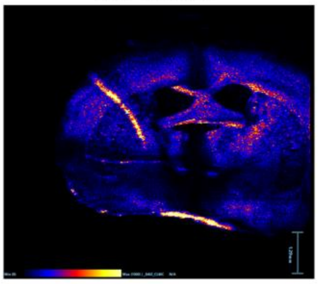

GlcCer(d18:1(8Z)/24:0(2OH[R]))
m/z 866.6544

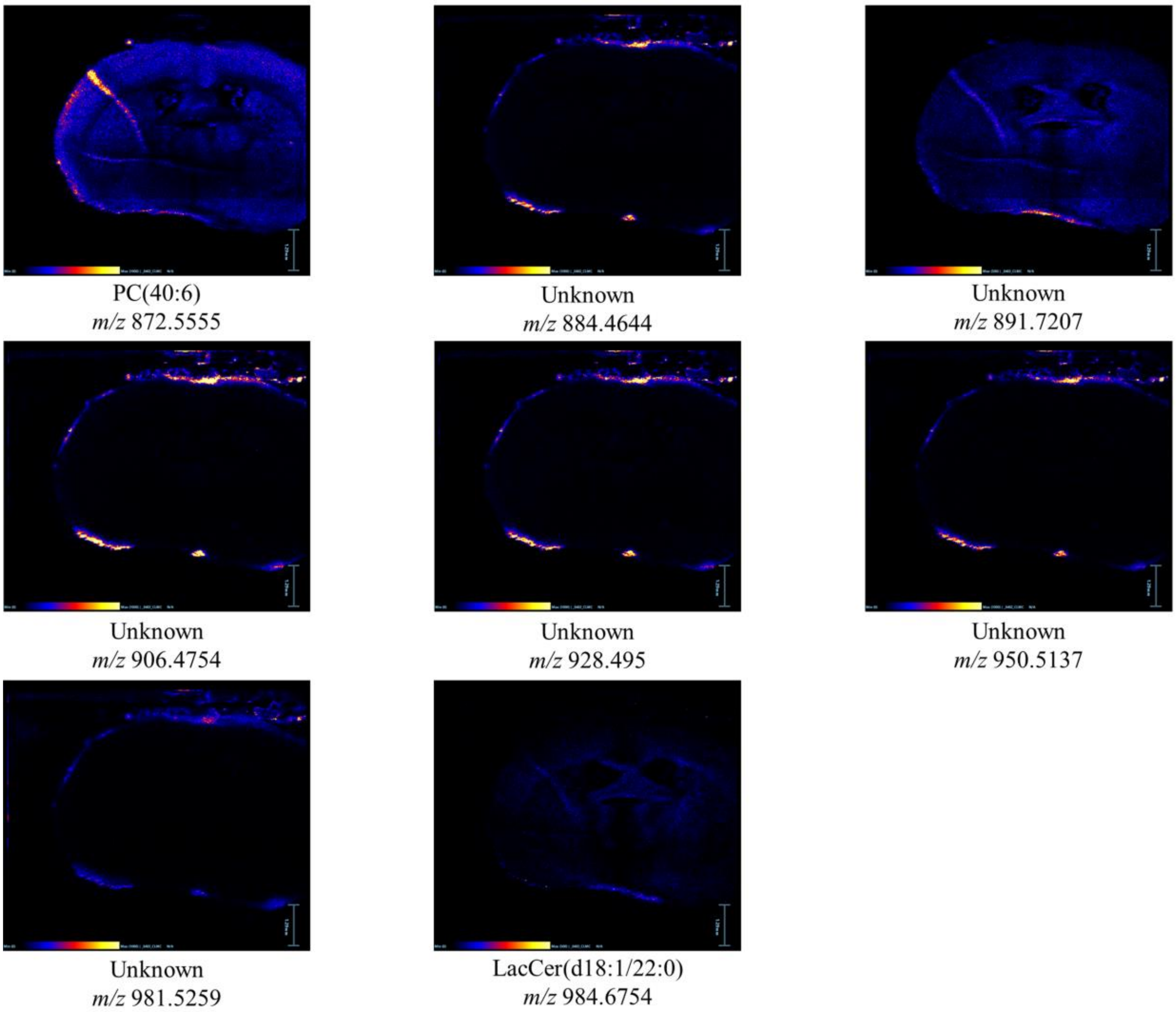

Figure S6. Mass spectrometry images of underivatized metabolites detected in mouse brain tissue with tentatively identified names.

Table S1. The BBII-derivatized metabolites detected in mouse brain tissue.

**Observed *m/z***	**Theoretical *m/z* with BBII derivatization**	**Theoretical *m/z* without BBII derivatization**	**Mass error (ppm)**	**Formula**	**Ionization type**	**Tentatively identified metabolite name**
320.1475	320.1452	92.0473	7.2	C3H8O3	$[M+BBII-2H_2O]^+$	Glycerol
408.1591	408.1613	180.0634	-5.4	C6H12O6	[M+BBII-2H2O]+	Glucose
420.1214	420.1249	192.0270	-8.3	C6H8O7	[M+BBII-2H2O]+	2,3-Diketo-L-gulonate
472.1665	472.1674	244.0695	-1.9	C9H12N2O6	[M+BBII-2H2O]+	Uridine
496.1761	496.1787	268.0808	-5.2	C10H12N4O5	[M+BBII-2H2O]+	Inosine
514.1929	514.1892	268.0808	7.2	C10H12N4O5	[M+BBII-H2O]+	Inosine
558.3709	558.3749	330.2770	-7.2	C19H38O4	[M+BBII-2H2O]+	MG(16:0)
584.3882	584.3906	356.2927	-4.1	C21H40O4	[M+BBII-2H2O]+	MG(18:1)
600.3884	600.3854	354.2770	5.0	C21H38O4	[M+BBII-H2O]+	MG(18:2)
632.4667	632.4633	386.3549	5.5	C27H46O	[M+BBII-H2O]+	Cholesterol
648.4605	648.4582	402.3498	3.5	C27H46O2	[M+BBII-H2O]+	25-Hydroxycholesterol
662.4391	662.4374	416.3290	2.6	C27H44O3	[M+BBII-H2O]+	7α,25-Dihydroxy-4-cholesten-3-one
678.4285	678.4324	432.3240	-5.7	C27H44O4	[M+BBII-H2O]+	3β,7α-Dihydroxy-5-cholestenoate
793.6437	793.6413	565.5434	3.0	C36H71NO3	[M+BBII-2H2O]+	Cer(d18:1/18:0)
971.6935	971.6890	725.5806	4.6	C42H79NO8	[M+BBII-H2O]+	GlcCer(d18:1/18:1)
999.7197						-

Table S2. The underivatized metabolites detected in mouse brain tissue.

Observed *m/z*	Theoretical *m/z*	Mass error (ppm)	Formula	Ionization type	Tentatively identified metabolite name
148.1117					-
162.1126	162.1125	0.616855579	C7H15NO3	M+H	L-Carnitine
184.0740	184.0739	0.543260071	C5H14NO4P	M+H	Phosphorylcholine
196.8779					-
212.8519					-
307.0461					-
429.2389					-
441.1920					-
483.0741					-
529.2530					-
543.9286					-
553.2753					-
558.6018					-
568.2874					-
573.2831					-
576.2816					-
587.9557					-
598.2929					-
602.6315					-
604.5038	604.5065	-4.466453214	C36H71NO3	M+K	Cer(d18:1/18:0)
606.3696					-
612.3127					-
617.3036					-
620.3079					-
642.3243					-
646.4417					-
656.3471					-
664.3317					-
678.3572					-
686.3513					-
700.3683					-
708.3658					-
713.4513	713.4518	-0.700818191	C42H67O8P	M+K	PA(34:1)
722.3842					-
730.3793					-
731.6051	731.6061	-1.366855744	C41H83N2O6P	M+H	SM(36:1)

734.5681	734.5694	-1.769744288	C40H80NO8P	M+H	PC(32:0)
739.4703					-
744.4954	744.4969	-2.014783406	C43H72NO8P	M+H-H2O	PE(38:7)
750.5543	750.5432	14.78928861	C43H76NO7P	M+H	PE(P-38:5)
752.3864					-
753.5876	753.5881	-0.663492431	C41H83N2O6P	M+Na	SM(36:1)
756.5518	756.5514	0.528714903	C40H80NO8P	M+Na	PC(32:0)
760.5886	760.5851	4.601720439	C42H82NO8P	M+H	PC(34:1)
769.5615	769.562	-0.64972023	C41H83N2O6P	M+K	SM(d18:1/18:0)
772.5243	772.5252	-1.165010539	C43H76NO7P	M+Na	PE(P-38:5)
774.4048					-
782.5618	782.5670	-6.644798465	C42H82NO8P	M+Na	PC(34:1)
784.5748	784.5827	-10.0690469	C42H84NO8P	M+Na	PC(34:0)
788.6197	788.6164	4.184543968	C44H86NO8P	M+H	PC(36:1)
790.5197					-
798.5400	798.5410	-1.252283853	C42H82NO8P	M+K	PC(34:1)
806.5119	806.5097	2.727803522	C43H78NO8P	M+K	PE(38:4)
808.5778	808.5851	-9.028115903	C46H82NO8P	M+H	PC(38:5)
810.6017	810.6007	1.233653018	C46H84NO8P	M+H	PC(38:4)
814.5346	814.5381	-4.296913797	C47H76NO8P	M+H	PE(42:9)
818.4323					-
820.5245	820.5253	-0.974985171	C44H80NO8P	M+K	PC(36:4)
822.6306	822.6220	10.45437637	C46H89NO8	M+K	GalCer(d18:1/22:0)
824.5599	824.5566	4.002150974	C44H84NO8P	M+K	PC(36:2)
826.5701	826.5723	-2.661594152	C44H86NO8P	M+K	PC(36:1)
830.5189	830.5097	11.07753468	C45H78NO8P	M+K	PE(40:6)
832.5701	832.5617	10.08934233	C46H84NO7P	M+K	PC(O-38:5)
836.6237	836.6164	8.725623834	C48H86NO8P	M+H	PC(40:5)
838.6259	838.6169	10.73195639	C46H89NO9	M+K	GlcCer(d18:1(8Z)/22:0(2OH[R]))
840.4378					-
844.5218	844.5253	-4.144340022	C46H80NO8P	M+K	PC(38:6)
848.6385	848.6376	1.060523361	C48H91NO8	M+K	GalCer(d18:1/24:1(15Z))
850.6550	850.6533	1.998464004	C48H93NO8	M+K	GalCer(d18:1/24:0)
863.6884	863.6976	-10.6518763	C49H97N2O6P	M+Na	SM(d18:1/26:1(17Z))
864.6420	864.6407	1.503514697	C46H89NO13	M+H	LacCer(d18:0/16:0)
866.6544	866.6482	7.153998589	C48H93NO9	M+K	GlcCer(d18:1(8Z)/24:0(2OH[R]))
872.5555	872.5566	-1.260663205	C48H84NO8P	M+K	PC(40:6)
884.4644					-

891.7207					-
906.4754					-
928.4950					-
950.5137					-
981.5259					-
984.6754	984.6748	0.60933823	C52H99NO13	M+K	LacCer(d18:1/22:0)
